# Metabolism is a major driver of hydrogen isotope fractionation recorded in tree‐ring glucose of *Pinus nigra*

**DOI:** 10.1111/nph.18014

**Published:** 2022-02-26

**Authors:** Thomas Wieloch, Michael Grabner, Angela Augusti, Henrik Serk, Ina Ehlers, Jun Yu, Jürgen Schleucher

**Affiliations:** ^1^ Department of Medical Biochemistry and Biophysics Umeå University 901 87 Umeå Sweden; ^2^ Institute of Wood Technology and Renewable Materials University of Natural Resources and Life Sciences Vienna 3430 Tulln an der Donau Austria; ^3^ Research Institute on Terrestrial Ecosystems National Research Council Porano (TR) 05010 Italy; ^4^ Department of Mathematics and Mathematical Statistics Umeå University 901 87 Umeå Sweden

**Keywords:** anaplerotic flux, Calvin–Benson cycle, change point, glucose‐6‐phosphate shunt, hydrogen stable isotopes, intramolecular isotope analysis, oxidative pentose phosphate pathway, sucrose‐to‐starch carbon partitioning

## Abstract

- Stable isotope abundances convey valuable information about plant physiological processes and underlying environmental controls. Central gaps in our mechanistic understanding of hydrogen isotope abundances impede their widespread application within the plant and biogeosciences.
- To address these gaps, we analysed intramolecular deuterium abundances in glucose of *Pinus nigra* extracted from an annually resolved tree‐ring series (1961–1995).
- We found fractionation signals (i.e. temporal variability in deuterium abundance) at glucose H^1^ and H^2^ introduced by closely related metabolic processes. Regression analysis indicates that these signals (and thus metabolism) respond to drought and atmospheric CO_2_ concentration beyond a response change point. They explain ≈ 60% of the whole‐molecule deuterium variability. Altered metabolism is associated with below‐average yet not exceptionally low growth.
- We propose the signals are introduced at the leaf level by changes in sucrose‐to‐starch carbon partitioning and anaplerotic carbon flux into the Calvin–Benson cycle. In conclusion, metabolism can be the main driver of hydrogen isotope variation in plant glucose.

## Introduction

Stable isotope measurements of the most abundant chemical elements in plants (H and C) convey valuable information about plant physiological and environmental processes. Plant archives, such as tree rings, preserve this information over millennia enabling elucidation of physiological and environmental dynamics that occur beyond the short time frames covered by manipulation or monitoring experiments (Loader *et al*., 2011; Peñuelas *et al*., 2011; Saurer *et al*., 2014; Frank *et al*., 2015; Köhler *et al*., 2016).

Among all stable isotopes, the hydrogen isotopes protium (^1^H) and deuterium (^2^H or D) exhibit the largest relative mass difference. As a result, hydrogen isotope effects are normally considerably larger than isotope effects of other elements (Melander & Saunders, 1980). Thus, from the physics point of view, hydrogen isotope compositions of plant compounds (commonly expressed in terms of *δ*D) have a remarkable potential to further our knowledge about plant physiological and environmental processes. However, current plant *δ*D models fail to predict the entire body of available empirical data (see later). Therefore, hydrogen isotopes, in contrast to carbon isotopes, have not yet evolved into standard tools within the plant and biogeosciences.

Current *δ*D models of plant organic matter exhibit three main components: (1) the *δ*D composition of plant water sources; (2) leaf water D enrichment; and (3) metabolic fractionations. (1) Plants take up soil water through roots. In most plants, this uptake and water transport through the xylem into leaves occur without detectable *δ*D shifts (Cernusak *et al*., 2016; Chen *et al*., 2020). Additionally, leaf water exchanges with atmospheric water vapour through stomata. Thus, leaf water inherits the *δ*D compositions of both soil water and atmospheric water vapour (Cernusak *et al*., 2016). (2) Stomatal evaporation causes D enrichment in leaf water. Adaptations of the Craig–Gordon model describe this process at the site of evaporation (Craig & Gordon, 1965; Flanagan *et al*., 1991; Farquhar *et al*., 2007). In a comprehensive series of gas exchange experiments over a range of isotopic and humidity conditions far exceeding natural conditions, Roden & Ehleringer (1999a) confirmed the robustness of a model that approximates leaf water D enrichment (Flanagan *et al*., 1991). Similarly, on an extensive *δ*D data set of leaf waters from very dry to very wet field sites, Kahmen *et al*. (2013) confirmed the robustness of the precise *δ*D enrichment model (Farquhar *et al*., 2007). Reanalysing this latter data set, Cernusak *et al*. (2016) reported a goodness of fit of *R* ^2^ = 0.92 (*n* = 332) between modelled and measured *δ*D data. These successful tests for robustness of leaf water *δ*D models strongly suggest that inconsistencies between modelled and measured *δ*D values of plant organic matter as reported by Waterhouse *et al*. (2002) derive from processes that occur downstream of leaf water D enrichment, that is from fractionating metabolic processes. (3) Yakir & DeNiro (1990) developed a *δ*D model describing fractionation in cellulose of aquatic plants and estimated metabolic fractionation factors of autotrophic and heterotrophic growth. To predict *δ*D values of tree‐ring cellulose, Roden *et al*. (2000) combined this model with a leaf water *δ*D model (Flanagan *et al*., 1991) and added a term accounting for *δ*D changes by partial reincorporation of hydrogen from xylem water during cellulose biosynthesis (=heterotrophic hydrogen exchange). Roden & Ehleringer (1999b, 2000) confirmed the robustness of their model for glasshouse‐ and field‐grown riparian trees with good access to water. A robustness test by Waterhouse *et al*. (2002) on tree rings of *Quercus robur* from a dry site failed. These latter authors suggested modelling metabolic fractionations as constants may be inadequate.

All hydrogen positions of leaf‐level metabolites inherit D fractionations present in leaf water, the hydrogen source of metabolism. By contrast, metabolic reactions cause D fractionations at specific intramolecular hydrogen positions that are directly or indirectly involved in the reaction mechanism (Schmidt *et al*., 2015). Such fractionations are known to result in pronounced *δ*D differences among hydrogen positions within a metabolite (Martin *et al*., 1986; Schleucher, 1998; Schmidt *et al*., 2003). Furthermore, it is now known that metabolic *δ*D variability can occur at a given intramolecular hydrogen position (Schleucher, 1998). Specifically, *δ*D variability at H^6^ of plant leaf glucose was explained by changes in the photorespiration‐to‐photosynthesis ratio (Schleucher, 1998; Ehlers *et al*., 2015). Moreover, growing six C_3_ species at ambient CO_2_ concentrations of 280 and 150 ppm, Cormier *et al*. (2018) reported whole‐molecule *δ*D differences of ≈ 20‰ (on average) in leaf α‐cellulose due to unknown fractionating metabolic processes. This shows the predictive abilities of plant *δ*D models will improve by accounting for variability in metabolic fractionations. To this end, however, underlying mechanisms need to be elucidated.

Based on findings by Waterhouse *et al*. (2002), we hypothesise metabolic processes manifest significant fractionation signals (throughout the paper, the term ‘signal’ denotes temporal variability in deuterium abundance) at carbon‐bound hydrogens in tree‐ring glucose under dry conditions. Since metabolic fractionations occur at specific hydrogen positions within molecules, the interpretability of conventional whole‐molecule data is fundamentally limited. For instance, not knowing the intramolecular location of metabolic fractionation impedes attempts to elucidate its enzymatic origin. Additionally, if located at a single hydrogen position, a *c.* 20‰ whole‐molecule effect scales up to *c.* 140‰ because glucose has seven H‐C positions. Therefore, to test our hypothesis, we measured intramolecular D abundance in tree‐ring glucose of *Pinus nigra* from a dry site in the Vienna region. This region was chosen for its comprehensive environmental monitoring. We first screen for metabolic fractionation signals. Then, we analyse signal–environment and signal–growth relationships and estimate the contribution of metabolic to whole‐molecule fractionation. Lastly, we discuss mechanisms of signal introduction and implications of our findings for isotope studies in the plant and biogeosciences.

## Materials and Methods

### Site and samples

*Pinus nigra* Arnold was sampled at Bierhäuselberg, Vienna region, Austria (48.13°N, 16.23°E, 350 m amsl). The site exhibits shallow, very dry soil and an open canopy with apparently low competition among trees (Leal *et al*., 2008). Sampling targeted dominant trees with umbrella‐shaped crowns indicating regular water shortage. From each of 19 specimens (age range: *c.* 92–156 yr), two 5‐mm stem cores were taken at breast height. Individual tree rings were dated by standard dendrochronological methods (Speer, 2010) and separated using a binocular microscope and a scalpel. To preclude growth‐related isotope signals, we analysed tree rings formed from 1961 to 1995 when all trees had reached a comparably stable canopy position. Across trees, all tree‐ring material of a given year was combined into annual pools yielding one sample per year. Thus, analytical results represent properties of the tree species at the site rather than individual trees.

### Intramolecular deuterium measurements

**Fig. 1:**
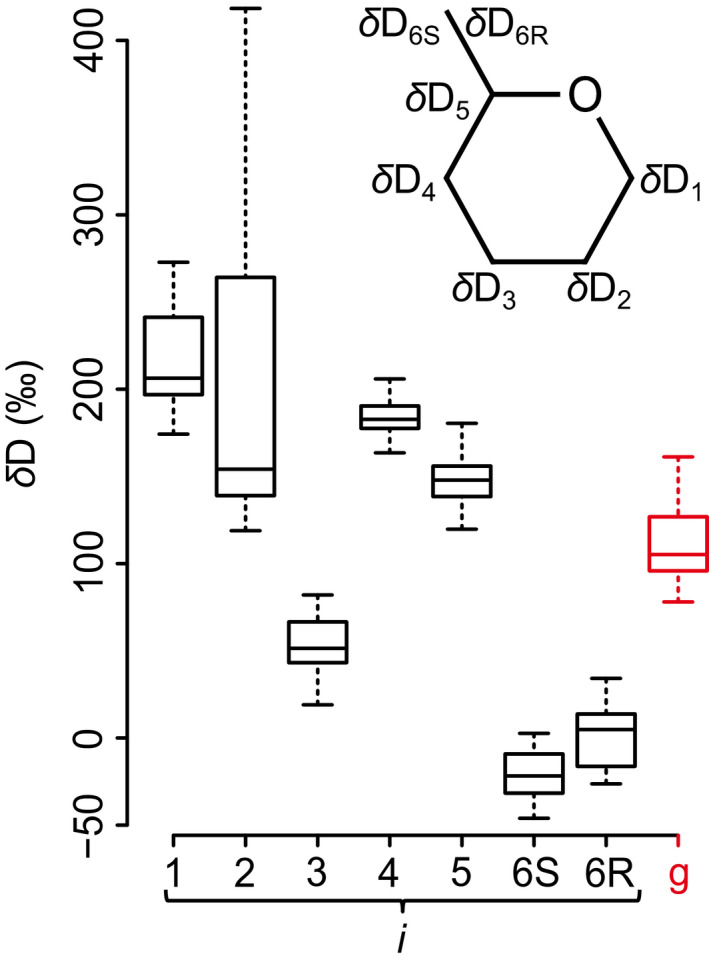
Variability in *δ*D time series shown by boxplots. *δ*D*_i_* and *δ*D_g_ denote time series of D abundance at intramolecular H‐C positions in tree‐ring glucose (black) and of the whole molecule (red), respectively. Data were acquired for tree‐ring glucose of *Pinus nigra* laid down from 1961 to 1995 at a site in the Vienna Basin (*δ*D*_i_*: ±SE = 5.4‰, *n* ≥ 3; *δ*D_g_: ±SE = 3.4‰, *n* ≥ 3). Outliers were removed before analysis (*δ*D_1_ to *δ*D_3_: *n* = 31; *δ*D_4_ and *δ*D_5_: *n* = 30; *δ*D_6S_: *n* = 26; *δ*D_6R_: *n* = 28; and *δ*D_g_: *n* = 25). Data reference: Average D abundance of the methyl group hydrogens of the glucose derivative used for NMR measurements. Insert: glucose carbon skeleton showing intramolecular locations of *δ*D*_i_* time series.

After sample randomisation, intramolecular D abundances of glucose were measured following a published protocol (Betson *et al*., 2006). Samples < 20 mg were excluded from analysis (1977, 1978, 1981 and 1982), because too much measurement time would have been required for sufficient precision. Quantitative D NMR spectra (*n* ≥ 3) were recorded using an AVANCE III 850 with a cryogenic probe optimised for D detection and ^19^F lock (Bruker BioSpin GmbH, Rheinstetten, Germany). Relative intramolecular D abundances were determined by signal deconvolution using Lorentzian line shape fits in TopSpin 4 (Bruker BioSpin GmbH, Rheinstetten, Germany). 3,6‐Anhydro‐1,2‐*O*‐isopropylidene‐α‐D‐glucofuranose has six methyl group hydrogens, which were introduced during derivatisation and were derived from a common batch of acetone. Their D abundance (arithmetic mean) was used as reference to compare glucose D abundances of tree‐ring samples from different years as follows:

(Eqn 1) $${\delta D}_{i}=\frac{D_{i}}{{\Sigma D}_{\mathrm{ME}}/6}‐1$$

with D*_i_* and D_ME_ denoting relative D abundance at glucose H‐C positions (Fig. 1) and the methyl group hydrogens, respectively.

### Environmental data

Monthly resolved data of precipitation, *PRE*, air temperature, *TMP*, sunshine duration, *SD*, global radiation, *RAD* and relative humidity were measured at the climate station Hohe Warte (WMO ID: 1103500). Air vapour pressure deficits, *VPD*, were calculated according to published procedures (Abtew & Melesse, 2013). We used monthly resolved data sets of the self‐calibrating Palmer drought severity index, *PDSI*, and the standardised precipitation–evapotranspiration index, *SPEI*, for 48.25N, 16.25E (Wells *et al*., 2004; Vicente‐Serrano *et al*., 2010). Annual atmospheric CO_2_ concentrations, *C*_a_, were measured at the Mauna Loa Observatory, Hawaii, by Pieter Tans (NOAA/ESRL, Boulder, CO, USA) and Ralph Keeling (Scripps Institution of Oceanography, La Jolla, CA, USA). Both the selected grid point and climate station are no more than a horizontal distance of 15 km from the sampling site with a negligible vertical offset. CO_2_ is well mixed in the atmosphere. Thus, all data should represent the site conditions well.

### Statistical analyses

We calculated the variance contribution of each intramolecular D time series, *δ*D*_i_*, to the whole‐molecule D time series, *δ*D_g_, as follows:

(Eqn 2) $${\delta D}_{g}=\frac{{\Sigma \delta D}_{i}}{7}$$

with the variance of *δ*D_g_ equal to the covariance average between *δ*D*_i_* and *δ*D_g_ as

(Eqn 3) $${\mathrm{Var}(\delta D}_{g})=\frac{{\Sigma \mathrm{Cov}(\delta D}_{i},{\delta D}_{g})}{7}$$

Thus, variance contributions of *δ*D*_i_* to *δ*D_g_ are given as

(Eqn 4) $${\mathrm{VC}}_{i}=\frac{{\mathrm{Cov}(\delta D}_{i},{\delta D}_{g})}{7{\times \mathrm{Var}(\delta D}_{g})}$$

See Supporting Information Notes S1 for information on other statistical analyses.

## Results

To better understand metabolic fractionations, we measured intramolecular D abundances at all seven H‐C positions of glucose extracted across an annually resolved *Pinus nigra* tree‐ring series (Fig. 1). Our time series cover the period from 1961 to 1995 but lack data for 1977, 1978, 1981 and 1982. Hence, the data set consists of seven intramolecular *δ*D*_i_* time series each comprising 31 observations (*n* = 7 × 31 = 217). Additionally, we derived the molecular average time series, *δ*D_g_ (*n* = 31).

### Outlier analysis

Outliers can potentially impair statistical analyses because they may reflect either experimental errors or extreme states of the system. Since we were interested in the normal functioning of plant metabolism and metabolic fractionation, we removed outliers to ensure robust statistical results. We identified outliers in *δ*D*_i_* time series (*n* = 31) as values that were 1.5 times the interquartile range below the first or above the third quartile. We removed one data point each from *δ*D_4_ and *δ*D_5_ (*n* = 30), three data points from *δ*D_6R_ (*n* = 28) and five data points from *δ*D_6S_ (*n* = 26) resulting in six missing data points in *δ*D_g_ (*n* = 25). The average standard errors of *δ*D*_i_* and *δ*D_g_ measurements are 5.4‰ and 3.4‰, respectively.

### *δ*D_1_ and *δ*D_2_ reflect highly variable and closely related metabolic processes

**Fig. 2:**
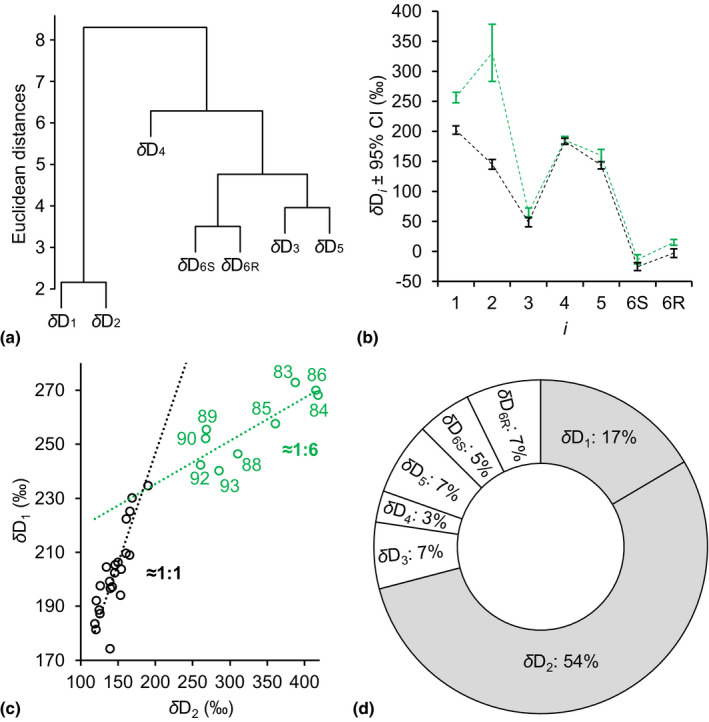
(a) Common variability in *δ*D*_i_* time series detected by hierarchical cluster analysis (HCA). (b) Intramolecular *δ*D*_i_* patterns of the low‐ and high‐value groups (black and green, respectively). (c) Relationship between *δ*D_1_ and *δ*D_2_ (overall *R* ^2^ = 0.93, *P* < 10^–15^, *n* = 31). Dotted lines: linear major axis regression models showing that *δ*D_1_ and *δ*D_2_ increase at a 1 : 1 ratio at low values (black) and at a 1 : 6 ratio at high values (green). (d) Per cent contributions of *δ*D*_i_* to the variance in *δ*D_g_. *δ*D*_i_* and *δ*D_g_ denote time series of D abundance at intramolecular H‐C positions in tree‐ring glucose and of the whole molecule, respectively. Data were acquired for tree‐ring glucose of *Pinus nigra* laid down from 1961 to 1995 at a site in the Vienna Basin (*δ*D*_i_*: ±SE = 5.4‰, *n* ≥ 3; *δ*D_g_: ±SE = 3.4‰, *n* ≥ 3). Outliers were removed before analysis (*δ*D_1_ to *δ*D_3_: *n* = 31; *δ*D_4_ and *δ*D_5_: *n* = 30; *δ*D_6S_: *n* = 26; *δ*D_6R_: *n* = 28; and *δ*D_g_: *n* = 25). Low‐ and high‐value groups were identified by HCA (Supporting Information Notes S2). Fig. 2(b) shows discrete data. Dashed lines were added to guide the eye. The variance partitioning analysis (2d) is based on years without missing data after removing outliers (*n* = 8 × 25). Data reference: Average D abundance of the methyl group hydrogens of the glucose derivative used for NMR measurements.

Intramolecular D abundances of *Pinus nigra* tree‐ring glucose differ among H‐C positions (Figs 1, 2b; Notes S2). Medial intramolecular differences exceed 225‰ (*δ*D_1_ vs *δ*D_6S_). This shows that metabolic fractionations, being position‐specific, have strong effects. Interestingly, in some years, intramolecular *δ*D differences approach 450‰ (Notes S2). Thus, metabolic fractionations are highly variable on the interannual scale.

Hierarchical cluster analysis (HCA) groups time series according to covariability. Time series carrying common signals, that is information about a common process or strongly related processes, form clusters. Performing HCA on our *δ*D*_i_* data set, we found a strong separation between a cluster comprising *δ*D_1_ and *δ*D_2_ and a cluster comprising *δ*D_3_ to *δ*D_6R_ (Fig. 2a). Additionally, we found separations within the *δ*D_3_ to *δ*D_6R_ cluster, with *δ*D_4_ showing the highest degree of independency. Thus, *δ*D*_i_* time series carry information about several fractionation processes.

While *δ*D_3_ to *δ*D_6R_ exhibit similar degrees of variability (Fig. 1; SD = ±10.5‰ to ±16.8‰, range = 42‰ to 63‰, *n* = 26–31), *δ*D_2_ varies considerably (SD = ±91.8‰, range = 300‰, *n* = 31) and *δ*D_1_ is also relatively variable (SD = ±28.4‰, range = 99‰, *n* = 31). Increased D variability at two out of seven H‐C positions indicates highly variable metabolic fractionations at these positions, H^1^ and H^2^.

To further investigate this, we performed HCA on annual *δ*D*_i_* patterns and found two groups (Notes S2). Fig. 2(b) shows the arithmetic average patterns of both groups. While *δ*D_3_ to *δ*D_6R_ values remain comparably constant under all conditions experienced by the trees, *δ*D_1_ and *δ*D_2_ separate annual *δ*D*_i_* patterns into a low‐value group (black, *n* = 22) and a high‐value group (green, *n* = 9). Average and median increases in *δ*D_1_ and *δ*D_2_ are statistically significant (one‐tailed Student’s t‐tests: *δ*D_1_, *P* < 10^–9^; *δ*D_2_, *p* < 10^–4^; one‐tailed Mood’s median tests: *δ*D_1_ and *δ*D_2_, *P* < 0.001). Since differences in *δ*D*_i_* patterns reflect differences in metabolism, this finding confirms that glucose H^1^ and H^2^ carry exceptionally variable metabolic fractionation signals. Underlying metabolic processes are closely related (Fig. 2a).

Nonmetabolic fractionation processes (e.g. leaf water D enrichment) have equal effects on all *δ*D*_i_*. In regression analysis investigating relationships among different *δ*D*_i_*, these processes can be expected to yield slopes = 1. By contrast, metabolic fractionation processes affect subsets of *δ*D*_i_* and may thus cause deviations from slope = 1. To investigate which kind of fractionation shapes the variation in the low‐ and high‐value groups, we performed major axis regression analysis on low and high *δ*D_1_ and *δ*D_2_ values (Fig. 2c, black and green circles, respectively). Compared with ordinary least squares regression analysis, major axis regression analysis yields truer parameter estimates when *x*‐variable data contain significant relative errors as is the case here for low‐value data. For low values, we found a slope of *c.* 0.81 with a 95% confidence interval of *c.* 0.60 to *c.* 1.07 (*n* = 22); that is, *δ*D_1_ and *δ*D_2_ increase at a *c.* 1 : 1 ratio. For high values, we found a slope of *c.* 0.16 with a 95% confidence interval of *c.* 0.07 to *c.* 0.25 (*n* = 9); that is, *δ*D_1_ and *δ*D_2_ increase at a *c.* 1 : 6 ratio. Thus, while *δ*D_1_ and *δ*D_2_ variability in the low‐value group is consistent with expectations related to nonmetabolic fractionation processes, high *δ*D_1_ and *δ*D_2_ values reflect additional effects by metabolic fractionation processes.

### Metabolism changes in response to dry conditions after a change point is crossed

To investigate the controls of the metabolic fractionation processes, we estimated metabolic fractionation at glucose H^1^ and H^2^ as

(Eqn 5) $$\varepsilon_{\mathrm{met}}=\frac{1/2\Sigma (D_{1},D_{2})}{1/5\Sigma (D_{3},D_{4},D_{5},D_{6S},D_{6R})}‐1$$

where D*_i_* denotes relative D abundance at glucose H‐C positions (Fig. 1). This procedure maximises metabolic fractionation signals located at H^1^ and H^2^, removes fractionation signals equally present at all H‐C positions (leaf water D enrichment) and minimises signals of processes affecting all H‐C positions similarly (heterotrophic hydrogen exchange). Weaker metabolic fractionation signals present at glucose H^3^ to H^6^ (Figs 1, 2a) will be further explored in future analyses of the data set.

In general, the fractionating metabolic processes may be under developmental control (Gray & Song, 1984). However, the formation of tree rings analysed here occurred when the trees had reached a stable canopy position. On average, tree‐ring width measurements of the samples reach back to 1865 (range: 1840–1918) and *δ*D*_i_* time series start at 1961. Thus, the fractionating metabolic processes can be expected to be controlled by environmental parameters. In the following, these are elucidated in six steps, which motivate two integrated isotope–environment models presented subsequently.

#### Step 1

Increases in *ε*_met_ are temporally confined to the second part of the study period (Fig. 1, 2, 1983–1993). This may indicate the presence of a response change point. To test this, we performed a batch change point analysis based on the nonparametric Mann–Whitney–Wilcoxon test (Ross, 2015). We found a change point at 1980 (*P* < 0.001, *n* = 31), showing that the observed frequency distribution of *ε*_met_ (Notes S3) does not align with the properties of a single theoretical probability distribution. That is, *ε*_met_ data of 1961–1980 and 1983 to 1995 follow different probability distributions (1981 and 1982 are missing). Thus, the *ε*_met_ time series exhibits a change point beyond which the fractionating metabolic processes are activated or upregulated.

#### Step 2

Upregulation of the fractionating metabolic processes occurs beyond a change point. Before the change point, environmental variability exerts no significant control (Fig. 1, 2, 1 : 1 ratio). To investigate when the change point is crossed, we correlated *ε*_met_ with different climate parameters. Since *ε*_met_ does not follow a normal distribution (Notes S3), we calculated Spearman’s rank correlation coefficient, *ρ*. Our analysis included four time periods: January to December, year; March to May, spring; March to November, growing season (Wieloch *et al*., 2018); and June to July, summer. For most periods, we found significant positive correlations of *ε*_met_ with *VPD* and significant negative correlations with *PDSI* and *SPEI* (Table 1). High *VPD*, low *PDSI* and low *SPEI* correspond to dry conditions. Thus, the results indicate that upregulation of the fractionating metabolic processes (high *ε*_met_) occurs in response to dry conditions.

**Table 1:** Spearman’s rank correlation coefficients and significance levels for relationships between *ε*_met_ and climate parameters within the period from 1961 to 1995 (*n* = 31).

	*VPD*	*PRE*	*PDSI*	*TMP*	*SD*	*RAD*	*C*_a_	*SPEI 1*	3	4	6	8	12	16	24	36	48
Year	0.40	0.01	−0.51	0.45	0.12	0.28	0.58	−0.21	−0.25	−0.27	−0.30	−0.37	−0.46	−0.47	−0.58	−0.60	−0.57
Spring	0.54	−0.13	−0.55	0.55	0.09	0.18	–	−0.24	−0.19	−0.15	−0.16	−0.29	−0.34	−0.43	−0.58	−0.54	−0.55
Growing season	0.39	−0.04	−0.50	0.33	0.01	0.24	–	−0.26	−0.21	−0.20	−0.29	−0.31	−0.43	−0.47	−0.59	−0.61	−0.56
Summer	0.28	−0.04	−0.49	0.02	−0.17	0.10	–	−0.09	−0.20	−0.21	−0.25	−0.31	−0.43	−0.41	−0.56	−0.65	−0.55

*ε*_met_ denotes the average deuterium fractionation by metabolic processes at glucose H^1^ and H^2^ (±SE = 3.5‰, *n* ≥ 3). Data for the calculation of *ε*_met_ were acquired for tree‐ring glucose of *Pinus nigra* laid down from 1961 to 1995 at a site in the Vienna Basin. Climate parameters: *VPD*, air vapour pressure deficit; *PRE*, precipitation; *PDSI*, the self‐calibrating Palmer drought severity index (Wells *et al*., 2004); *TMP*, air temperature; *SD*, sunshine duration; *RAD*, global radiation; *C*_a_, atmospheric CO_2_ concentrations; *SPEI*, the standardised precipitation–evapotranspiration index calculated for different timescales (1–48 months) (Vicente‐Serrano *et al*., 2010). Integration periods for the calculation of climate parameters: January to December, year; March to May, spring; March to November, growing season (Wieloch *et al*., 2018); and June to July, summer. Significance levels: < 0.05, light blue/light pink; < 0.01, blue/pink; and < 0.001, dark blue/dark pink.

#### Step 3

*SPEI* can be calculated for different timescales indicating different types of hydrological drought (Vicente‐Serrano *et al*., 2010). While short timescales indicate variability in soil moisture, long timescales indicate variability in groundwater storage (Vicente‐Serrano *et al*., 2010). To investigate the effects of different drought types on the fractionating metabolic processes, we correlated *ε*_met_ with *SPEI* calculated for timescales of 1, 3, 4, 6, 8, 12, 16, 24, 36 and 48 months. We found increasing correlation coefficients from short to long timescales (Table 1), indicating that long‐term drought promoted upregulations of the fractionating metabolic processes (possibly by groundwater depletion). Long‐term drought events have a low temporal frequency (Vicente‐Serrano *et al*., 2010). This may explain why upregulations of the fractionating metabolic processes occur not scattered over the entire study period but over consecutive years during the second half of the study period (Fig. 2c).

#### Step 4

In addition to correlations indicative of drought control, we found a significant positive correlation between *ε*_met_ and *C*_a_ (Table 1), which may constitute a pseudocorrelation resulting from intercorrelation between *C*_a_ and long‐term drought (Pearson’s correlation between *C*_a_ and the growing‐season 48‐month *SPEI*: *r* = −0.83, *P* < 10^–7^, *n* = 31). For the year and spring, we found significant positive correlations between *ε*_met_ and *TMP* (Table 1). Thus, air temperature may contribute to upregulations of the fractionating metabolic processes. Alternatively, the *TMP* correlation may be explained by intercorrelation with drought parameters. Overall, long‐term drought shows the highest correlation coefficients (Table 1), indicating that it is the most important environmental control. Furthermore, we considered pathogens as a cause for upregulations of the fractionating metabolic processes. The Research Centre for Forests (Vienna, Austria) closely monitors and reports on pathogen status. To our knowledge, there are no reports of major damage by pathogens for the studied *Pinus nigra* stand during the 1980s and the early 1990s. Thus, significant effects of pathogens on upregulations of the fractionating metabolic processes are unlikely. Similarly, effects by forest management interventions are unlikely since the stand is not used economically (Leal *et al*., 2008). Effects by air pollutants could not be assessed due to limited data availability.

#### Step 5

**Fig. 3:**
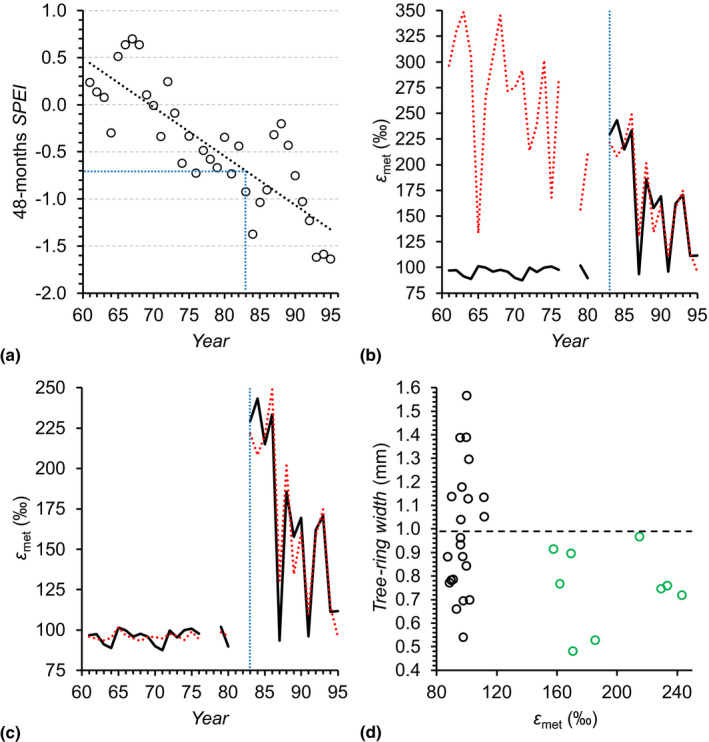
(a) Long‐term development of growing‐season drought in the study region. Trendline analysis shows a significant increase in drought severity with shifts from ‘nondrought’ from 1961 to 1978, to ‘mild drought’ from 1979 to 1988, to ‘moderate drought’ from 1989 to 1995 (dotted black line, *R* ^2^ = 0.69, *P* < 10^–9^, *n* = 35). *SPEI* denotes the standardised precipitation–evapotranspiration index (Vicente‐Serrano *et al*., 2010). Here, it was calculated for the growing season (March to November (Wieloch *et al*., 2018)) at 48.25°N, 16.25°E on a 48‐month timescale indicative of long‐term drought variability (black circles). Expressions of SPEI values indicate the drought severity as ≥−0.49, nondrought; −0.5 to −0.99, mild drought; −1 to −1.49, moderate drought; −1.5 to −1.99, severe drought; and <−2, extreme drought (McKee *et al*., 1993; Vicente‐Serrano *et al*., 2010). (b, c) Comparison of measured (solid black line) and modelled (dotted red line) metabolic fractionation at glucose H^1^ and H^2^, *ε*_met_ (*n* = 31). In (b) and (c), data were modelled by a multivariate linear model (Eqn 6) and a multivariate change point model (Eqn 7; Table 3) (Fong *et al*., 2017), respectively. Dotted blue line: response change point as determined by the change point model. (d) Relationship between *ε*_met_ and tree‐ring width. Green and black circles: years in which the fractionating metabolic processes were upregulated and downregulated, respectively (Fig. 2). Dashed line: average tree‐ring width of years in which the fractionating metabolic processes were downregulated (black circles, 0.99 mm). Tree‐ring widths were measured for previous dendro‐ecological studies (Leal *et al*., 2008). Data for the calculation of *ε*_met_ were acquired for tree‐ring glucose of *Pinus nigra* laid down from 1961 to 1995 at a site in the Vienna Basin (±SE = 3.5‰, *n* ≥ 3).

To assess the magnitude of the drought shift indicated by correlation, we plotted the growing‐season 48‐month *SPEI* as function of time (Fig. 3a). Trendline analysis shows a significant increase in drought severity with a long‐term shift from ‘nondrought’ from 1961 to 1978, to ‘mild drought’ from 1979 to 1988, to ‘moderate drought’ from 1989 to 1995 according to the classification of drought severity (*R* ^2^ = 0.69, *P* < 10^–9^, *n* = 35) (McKee *et al*., 1993; Vicente‐Serrano *et al*., 2010). Thus, upregulations of the fractionating metabolic processes in between 1983 and 1993 occurred when the study region experienced mild‐to‐moderate drought. Note that actual conditions at the site may have deviated from regional conditions plotted in Fig. 3(a).

#### Step 6

In 1994 and 1995, long‐term drought was exceptionally strong (Fig. 3a). However, this did not result in significant upregulations of the fractionating metabolic processes (Fig. 2c). Therefore, we hypothesise long‐term drought beyond a critical level is merely setting the stage for upregulation and secondary climate factors exert control on shorter timescales. To investigate this, we repeated the *ε*_met_–climate correlation analysis with data of the second half of the study period, 1983 to 1995. We justify this data selection as follows. In 1983, the upregulation of the fractionating metabolic processes occurred for the first time (Fig. 2c). From 1983 to 1995, long‐term drought became more severe (Fig. 3a, dotted black line). Thus, the site conditions during this period may have been generally favourable for upregulation. Before 1983, long‐term drought was less severe and may have rendered upregulations impossible (Fig. 3a). In this scenario, control by secondary climate factors would have been impossible and considering their variability would impair correlation results. Since the selected subset of *ε*_met_ (1983–1995) follows a normal distribution (Notes S3), we calculated Pearson’s correlation coefficients, *r*. We found significant negative correlations between *ε*_met_ and *PRE* (Table 2). Under generally dry conditions, low precipitation may result in low soil moisture. Thus, the results indicate that upregulations of the fractionating metabolic processes (high *ε*_met_) occurred in response to short‐term depletions in soil moisture when long‐term drought was beyond the critical level. In this respect, it is noteworthy that *Pinus nigra* reportedly has a well‐developed lateral root system in upper soil layers to access soil water but can additionally tap into deep water sources if available (Stokes *et al*., 2002; Peñuelas & Filella, 2003). Furthermore, we found a significant negative correlation between *ε*_met_ and annual *C*_a_ (Table 2). This correlation is not explained by intercorrelation between *C*_a_ and long‐term drought (Pearson’s correlation between *C*_a_ and the growing‐season 48‐month SPEI: *r* = −0.39, *P* > 0.1, *n* = 13). Thus, the result indicates that high atmospheric CO_2_ concentrations counteracted upregulations of the fractionating metabolic processes (high *ε*_met_) when long‐term drought was beyond the critical level.

**Table 2:** Pearson’s correlation coefficients and significance levels for relationships between *ε*_met_ and climate parameters within the period from 1983 to 1995 (*n* = 13).

	*VPD*	*PRE*	*PDSI*	*TMP*	*SD*	*RAD*	*C*_a_	*SPEI 1*	3	4	6	8	12	16	24	36	48
Year	0.26	−0.69	−0.51	−0.17	−0.12	−0.34	−0.73	−0.18	−0.20	−0.24	−0.28	−0.28	−0.10	0.03	0.03	−0.04	0.04
Spring	0.52	−0.71	−0.29	0.23	−0.08	−0.19	–	−0.28	−0.01	0.02	−0.08	−0.13	0.20	0.27	0.10	0.07	0.22
Growing season	0.27	−0.67	−0.52	−0.14	0.01	−0.33	–	−0.27	−0.17	−0.20	−0.29	−0.29	−0.18	0.03	0.02	−0.04	0.03
Summer	0.17	−0.67	−0.54	−0.47	−0.30	−0.51	–	−0.39	−0.44	−0.46	−0.31	−0.35	−0.29	−0.05	−0.03	−0.10	0.05

*ε*_met_ denotes the average deuterium fractionation by metabolic processes at glucose H^1^ and H^2^ (±SE = 3.4‰, *n* ≥ 3). Data for the calculation of *ε*_met_ were acquired for tree‐ring glucose of *Pinus nigra* laid down from 1983 to 1995 at a site in the Vienna Basin. Climate parameters: *VPD*, air vapour pressure deficit; *PRE*, precipitation; *PDSI*, the self‐calibrating Palmer drought severity index (Wells *et al*., 2004); *TMP*, air temperature; *SD*, sunshine duration; *RAD*, global radiation; *C*_a_, atmospheric CO_2_ concentrations; *SPEI*, the standardised precipitation–evapotranspiration index calculated for different timescales (1–48 months) (Vicente‐Serrano *et al*., 2010). Integration periods for the calculation of climate parameters: January to December, year; March to May, spring; March to November, growing season (Wieloch *et al*., 2018); and June to July, summer. Significance levels: < 0.05, light blue; < 0.01, blue.

#### Model 1

To further corroborate the existence of a response change point (Step 1), and the requirement of long‐term drought (Steps 2 and 3) for the manifestation of effects by secondary climate factors (Step 6), we modelled two *ε*_met_ groups as functions of *PRE* and *C*_a_ as follows:

(Eqn 6) $$\varepsilon_{\mathrm{met}}=\alpha_{1}+\alpha_{2}\mathrm{PRE}+\alpha_{3}C_{a}$$

While the first group includes data from 1961 to 1982 corresponding to less severe long‐term drought, the second group includes data from 1983 to 1995 corresponding to more severe long‐term drought (Fig. 3a). For the latter group, we found adequate linear models including, as explanatory variables, spring *PRE* and annual *C*_a_ (*R* ^2^ = 0.74, *P* < 0.01, *n* = 13), and summer *PRE* and annual *C*_a_ (*R^2^* = 0.78, *P* < 0.001, *n* = 13). The most adequate model we found includes summed spring and summer *PRE* and annual *C*_a_ (*R* ^2^ = 0.87, *P* < 10^–4^, *n* = 13, *α*_1_ = 1801.97, *α*_2_ = −0.566, *α*_3_ = 4.1851; see Notes S4 for bivariate relationships of *ε*_met_ with *PRE* and *C*_a_, respectively). Both *PRE* and *C*_a_ contributed significantly to all models (*P* < 0.05). For *ε*_met_ data of the first group, 1961 to 1982, the most adequate model has no explanatory power (*P* > 0.1, *n* = 18). Modelling *ε*_met_ by the most adequate model works well for the second group, 1983–1995, yet produces large offsets between all measured and modelled values of the first group (Fig. 3b). This corroborates that (1) a response change point exists and (2) the manifestation of effects by secondary climate factors requires long‐term drought. Furthermore, it should be noted that the most adequate model accounts well for low *ε*_met_ values in 1994 and 1995 (Fig. 3b). During these years, long‐term drought was exceptionally severe, yet PRE and *C*_a_ were high (Fig. 3a; Notes S4). Thus, long‐term drought alone may not necessarily lead to upregulation of the fractionating metabolic processes. High precipitation during spring and summer, as well as high atmospheric CO_2_ concentrations, may exert counteracting effects.

#### Model 2

Above, we found indications for several properties of *ε*_met_ of *Pinus nigra* tree‐ring glucose. To test these properties in an integrated way and to estimate the change point value, we fitted a change point model to the entire dataset (Fong *et al*., 2017). It takes the form

(Eqn 7) $$\varepsilon_{\mathrm{met}}=\beta_{1}+\beta_{2}z_{1}+\beta_{3}z_{2}+\beta_{4}I\left(x>e\right)+\beta_{5}z_{1}I\left(x>e\right)+\beta_{6}z_{2}I\left(x>e\right)$$

where *β*_1_ to *β*_6_ are model coefficients, *z*_1_ is March‐to‐June *PRE*, *z*_2_ is annual *C*_a_, *x* is the trend of the growing‐season 48‐month *SPEI* used as change point variable (Fig. 3a, dotted black line), and e is the change point value. If *x* > e, then *I*(*x* > e) equals 1, else *I*(*x* > e) equals 0. The fitted model explains 94% of the variability in *ε*_met_ and is highly significant (*P* < 10^–15^, *n* = 31). All explanatory variables and the change point contribute significantly (Table 3). The estimated change point is at −0.7 ± 0.05SE corresponding to mild long‐term drought (*P* < 10^–15^, Table 3; Fig. 3a). The presence/absence and directionality of modelled *ε*_met_ relationships with precipitation and atmospheric CO_2_ concentration agree with findings given above (Tables 1, 2, 3). In contrast to the model without response change point (Fig. 3b), the change point model captures the variability in the entire *ε*_met_ data set (Fig. 3c). Thus, this integrated change point model corroborates the following findings. First, *ε*_met_ exhibits a response change point. Below this change point, *ε*_met_ is largely constant with values of *c.* 96‰. Above the change point, *ε*_met_ variability is high. Second, change point crossing may require long‐term drought beyond a critical level. Third, March‐to‐July precipitation and annual atmospheric CO_2_ concentration may govern *ε*_met_ variability in short timescales when long‐term drought is beyond the critical level. Note, all relationships indicated by modelling require experimental confirmation in controlled settings.

**Table 3:** Estimated coefficients of the *ε*_met_ change point model.

Coefficient	Estimate	SE*	Lower 95% CI	Upper 95% CI	*P*‐value*
*β*_1_	1801.97	479.02	863.10	2740.85	<0.001
*β*_2_	−0.56599	0.17577	−0.91051	−0.22147	<0.01
*β*_3_	−4.1851	1.4164	−6.9613	−1.4090	<0.01
*β*_4_	−1722.11	479.90	−2662.71	−781.51	<0.001
*β*_5_	0.58782	0.17749	0.23993	0.93571	<0.001
*β*_6_	4.2138	1.4574	1.3573	7.0703	<0.01
e	−0.70310	0.05296	−0.80690	−0.59930	<10^–15^

A change point model was fitted to measured *ε*_met_ data (*R* ^2^ = 0.94, *P* < 10^–15^, *n* = 31, Eqn 7) (Fong *et al*., 2017). *ε*_met_ denotes the average deuterium fractionation by metabolic processes at glucose H^1^ and H^2^ (±SE = 3.5‰, *n* ≥ 3). Data for the calculation of *ε*_met_ were acquired for tree‐ring glucose of *Pinus nigra* laid down from 1961 to 1995 at a site in the Vienna Basin. *β*_1_ to *β*_6_, and e denote model coefficients (Eqn 7). SE and CI denote the standard error and confidence interval, respectively. Asterisks mark estimations, which assume that bootstrap sampling followed a normal distribution.

### Variability in *δ*D_g_ is predominantly controlled by fractionating metabolic processes

Metabolic fractionations in *δ*D_1_ and *δ*D_2_ have a strong weight on whole‐molecule D variability, *δ*D_g_. Variance partitioning shows that *δ*D_1_ and *δ*D_2_ together account for 71% of the variance in *δ*D_g_ (Fig. 2d). By contrast, *δ*D_3_ to *δ*D_6R_ each accounts for 5.8% on average. Assuming the variability in *δ*D_3_ to *δ*D_6R_ reflects the combined influence of nonmetabolic fractionation processes affecting all *δ*D*_i_*, such as leaf water D enrichment, metabolic fractionations in *δ*D_1_ and *δ*D_2_ together account for 59.4% of the variance in *δ*D_g_ (71%–2 × 5.8%). When years affected by metabolic fractionation are considered exclusively (green dots in Fig. 2c), metabolic fractionations in *δ*D_1_ and *δ*D_2_ together account for 74.2% of the variance in *δ*D_g_ (Notes S5). After excluding the period affected by metabolic fractionation from the analysis (1983–1995), all *δ*D*_i_* exhibit similar degrees of variance and contribute similar to *δ*D_g_ (Notes S6) consistent with expectations related to nonmetabolic fractionation processes. In conclusion, here, *δ*D_g_ variability is predominantly controlled by fractionating metabolic processes unaccounted for in current models. Note, our estimations implicitly assume invariability of metabolic fractionations in *δ*D_3_ to *δ*D_6R_. This simplifies actual conditions and results in underestimation of the influence of metabolic fractionations on *δ*D_g_.

### Altered metabolism is associated with below‐average yet not exceptionally low growth

Metabolic changes as reflected by changes in metabolic fractionation may impact on growth. To test this, we investigated the relationship between *ε*_met_ and tree‐ring width. We found that when the fractionating metabolic processes were upregulated, significantly more narrow tree rings were formed compared with those when they were downregulated (Fig. 3d, one‐tailed *t*‐test: green vs black circles, 0.75 vs 0.99 mm, *P* < 0.05, *n* = 9 and 22). Thus, altered metabolism is associated with below‐average growth. However, equally narrow tree rings were formed in > 50% of the years in which the fractionating metabolic processes were downregulated (Fig. 3d; tree‐ring widths < 0.99 mm, *n* = 12). Thus, upregulations of the fractionating metabolic processes are not associated with exceptional growth declines.

## Discussion

### *ε*_met_: An isotope biomarker reports metabolic changes and drought

Here, we found highly variable metabolic fractionation signals at glucose H^1^ and H^2^ (Figs 1, 2). We approximated these signals by calculating *ε*_met_ (Eqn 5) and propose *ε*_met_ is a sensitive isotope biomarker reflecting changes in plant metabolism. Furthermore, we found a change point in *ε*_met_ (Step 1; Fig. 3a–c) suggesting *ε*_met_ features a two‐state property. This property promises unambiguous identification of alternate metabolic states: a stable state where the fractionating metabolic processes remain downregulated, and a state where these processes are upregulated (upon crossing a change point). By contrast, other biomarkers develop in a purely linear manner with indistinct transitions between states hindering attempts to ascertain plant functioning. While the mechanisms behind other biomarkers in plant archives often remain elusive, *ε*_met_ can be (1) linked to specific physiological processes and (2) incorporated into fractionation models (see later). Upon process elucidation, *ε*_met_ may help to find plants with the make‐up to support crucial ecosystem services such as food and resource security.

As indicated by modelling, atmospheric CO_2_ concentrations and drought exert control over *ε*_met_ in *Pinus nigra* studied here (Tables 1, 2, 3; Fig. 3a–c). Values of *ε*_met_ > 105‰ indicate long‐term drought events affecting plant metabolism (Fig. 3a–c). Given careful sample selection (e.g. designed to avoid bias related to tree age), this may enable identification of historical occurrences. Fitting a model to *ε*_met_ values > 105‰ (Fig. 3b, *ε*_met_ = 1802–0.566*PRE*‐4.185*C*_a_) may enable analyses of metabolic effects of short‐term drought and *C*_a_ during long‐term drought events (by interpretation of the respective model terms), and reconstructions of short‐term drought events during long‐term drought events (by solving for *PRE* with historical *C*_a_ data available).

*Pinus nigra* can access water from upper soil layers and from deep water sources (Stokes *et al*., 2002; Peñuelas & Filella, 2003). Accordingly, at our study site, metabolic responses apparently require long‐term drought (possibly involving groundwater depletion). In the absence of long‐term drought, *ε*_met_ is blind to variability in short‐term drought (possibly involving low soil moisture) and *C*_a_ (Fig. 3a–c). By contrast, species without groundwater access may provide this information. Lastly, *ε*_met_ may not always report drought, but it may always report source‐limited growth conditions (cf. Wieloch *et al*., 2022a).

### The role of leaf intercellular CO_2_ concentrations

As indicated by modelling, upregulations of the fractionating metabolic processes occur in response to drought, yet high atmospheric CO_2_ concentrations exert counteracting effects (Tables 1, 2, 3; Fig. 3a–c). Isohydric plant species such as *Pinus nigra* respond to drought by closing their stomata (Sade *et al*., 2012). This impedes CO_2_ uptake and promotes low intercellular CO_2_ concentrations, *C*_i_. By contrast, increasing *C*_a_ promotes increasing *C*_i_. Thus, upregulations of the fractionating metabolic processes may be mediated by low *C*_i_. In principle, whole‐molecule stable carbon isotope ratios of plant organic matter, *δ* ^13^C, enable *C*_i_ estimations because ^13^C fractionation during carbon uptake is related to *C*_i_/*C*_a_ (Farquhar *et al*., 1982; Evans *et al*., 1986). However, in the samples studied here, processes that are independent of carbon uptake fractionation control *δ* ^13^C at three out of six glucose carbon positions (Wieloch *et al*., 2018, 2021b, 2022b). Therefore, we decided against using *δ* ^13^C‐derived *C*_i_ estimates to test this hypothesis.

### Stable isotope methodology

Variability in *δ*D_g_ is predominantly controlled by metabolic fractionation at glucose H^1^ and H^2^ (Fig. 2d). Since *δ*D_g_ can be measured by high‐throughput isotope ratio mass spectrometry, a technique accessible to numerous laboratories, we investigated possibilities to (1) detect *δ*D_g_ data sets affected by metabolic fractionation; (2) separate *δ*D_g_ data points affected by metabolic fractionation from other data points; and (3) retrieve information from *δ*D_g_ about metabolic fractionation (Notes S7).

We found our *δ*D_g_ data set holds clues to effects by metabolic fractionation at glucose H^1^ and H^2^ (Notes S7). Furthermore, the separation of *δ*D_g_ data points affected by metabolic fractionation from other data points seems feasible yet not with high confidence. Discarding data sets/data points affected by metabolic fractionation at glucose H^1^ and H^2^ may improve *δ*D_g_ analyses of nonmetabolic fractionation processes. However, analytical results will be impaired by other metabolic fractionations.

Modelling *ε*_met_, we found both a response change point marking the onset of a period with conditions favourable for upregulation of metabolic fractionation processes and environmental dependences of upregulation (Table 3; Eqn 7; Fig. 3c). Applying this model to *δ*D_g_, we found the same change point, but the environmental dependences of upregulation were not sufficiently constrained for interpretation (Notes S7). Thus, by itself, *δ*D_g_ analysis of glucose does not yield robust information about the environmental dependences of metabolic fractionation at H^1^ and H^2^. However, in combination with modelled or measured leaf water *δ*D values, information on overall metabolic fractionation may be retrieved (cf. Cormier *et al*., 2018). By contrast, resolving information about different metabolic fractionations requires the intramolecular approach.

Current *δ*D_g_ models describe metabolic fractionations as being constant (Roden *et al*., 2000). As shown here, this is inadequate for plants grown under dry conditions where metabolic fractionations can exert predominant control over *δ*D_g_ variability (Fig. 2d). Thus, to model plant D abundance over the whole range of environmental conditions, metabolic fractionations need to be incorporated as variables, which requires detailed knowledge about underlying processes.

Metabolic fractionation processes known to affect tree‐ring glucose H^1^ and H^2^ cannot explain our findings. Specifically, *Picea abies* reportedly exhibits similar heterotrophic hydrogen exchange rates at tree‐ring glucose H^1^ and H^2^ (*c.* 42%) (Augusti *et al*., 2006). Since *Pinus nigra* as a close relative can be expected to behave similarly, heterotrophic hydrogen exchange at these positions cannot cause pronounced deviations from slope = 1 as observed for the *δ*D_1_‐*δ*D_2_ regression (Fig. 2c). Therefore, we will now derive experimentally testable theories on the metabolic origins of the fractionation signals reported here.

### Theory 1. Isotope fractionation related to sucrose‐to‐starch carbon partitioning

**Fig. 4:**
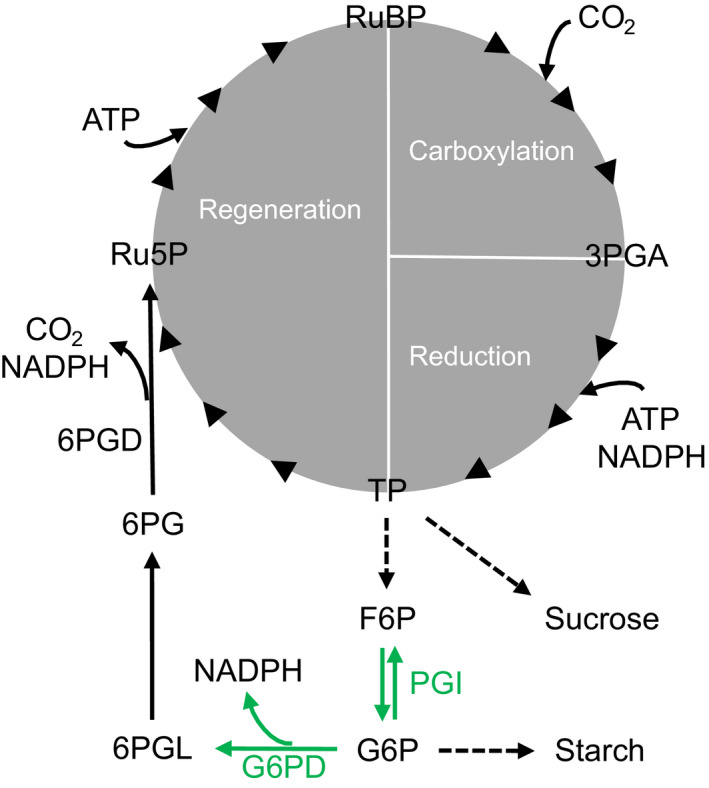
Sucrose‐to‐starch carbon partitioning and anaplerotic carbon flux into the Calvin–Benson cycle. Green: enzyme reactions proposedly introducing D isotope signals at glucose H^1^ and H^2^. G6PD has a kinetic isotope effect of *α*_D_ = *k*_H_/*k*_D_ = 2.97 (Hermes *et al*., 1982), while PGI has a kinetic isotope effect of 2.22 in the F6P to G6P direction and an equilibrium isotope effect of 1.11 in G6P (Rose & O’Connell, 1961; Wieloch *et al*., 2022a). Dashed arrows: intermediate reactions not shown. Enzymes: 6PGD, 6‐phosphogluconate dehydrogenase; G6PD, glucose‐6‐phosphate dehydrogenase; and PGI, phosphoglucose isomerase. Metabolites: 3PGA, 3‐phosphoglycerate; 6PG, 6‐phosphogluconate; 6PGL, 6‐phosphogluconolactone; ATP, adenosine triphosphate; F6P, fructose 6‐phosphate; G6P, glucose 6‐phosphate; NADPH, nicotinamide adenine dinucleotide phosphate; Ru5P, ribulose 5‐phosphate; RuBP, ribulose 1,5‐bisphosphate; and TP, triose phosphates (glyceraldehyde 3‐phosphate, dihydroxyacetone phosphate).

We found a highly variable metabolic fractionation signal at tree‐ring glucose H^2^ (*ε*_met_ at H^2^: SD *c.* ±79‰, range *c.* 260‰, *n* = 31), which occurs upon the crossing of a response change point (Fig. 3a–c). At the leaf level, tree‐ring glucose has two precursors, starch and sucrose. Growing *Phaseolus vulgaris* and *Spinacia oleracea* under optimal conditions, Schleucher *et al*. (1999) reported D depletions at glucose H^2^ of leaf starch relative to sucrose of 333‰ and 500‰, respectively. This difference may contribute to the signal at tree‐ring glucose H^2^ when the relative flux of assimilated carbon into starch and sucrose changes (Fig. 4). Sucrose‐to‐starch carbon partitioning ratios are controlled primarily by the rate of carbon assimilation (Sharkey *et al*., 1985) and were shown to increase with decreasing assimilation rates (Sharkey *et al*., 1985), decreasing *C*_i_ (Sharkey *et al*., 1985), decreasing light (Sharkey *et al*., 1985; Quick *et al*., 1989) and increasing drought (Quick *et al*., 1989, 1992; Vassey & Sharkey, 1989). Reported responses are functionally coherent and qualitatively consistent across all species studied. Furthermore, responses of sucrose‐to‐starch carbon partitioning to ecophysiological changes exhibit change points. For instance, in leaves of *Phaseolus vulgaris*, the sucrose‐to‐starch carbon partitioning ratio was found to be largely constant at *C*_i_ ≥ 150 ppm yet increases below this change point from *c.* 0.5 to *c.* 4 (Sharkey *et al*., 1985). Thus, as *C*_i_ drops below the change point, the relative contribution of leaf sucrose to tree‐ring glucose biosynthesis may increase, and tree‐ring glucose H^2^ may gradually approach the higher relative D abundance of leaf sucrose.

### Theory 2. Isotope fractionation related to anaplerotic carbon flux into the Calvin–Benson cycle

Metabolic fractionation signals at tree‐ring glucose H^1^ and H^2^ are closely related (Fig. 2a,c). We propose this is due to (1) isotope fractionation related to anaplerotic carbon flux into the Calvin–Benson cycle (CBC) affecting both positions; and (2) dependences of both anaplerotic carbon flux into the CBC and sucrose‐to‐starch carbon partitioning on the rate of carbon assimilation.

Sharkey & Weise (2016) proposed the CBC is anaplerotically refilled by injection of five‐carbon sugar phosphates from the stromal pentose phosphate pathway with glucose 6‐phosphate (G6P) as precursor (Fig. 4). Primary flux control is believed to be exerted at the level of stromal phosphoglucose isomerase (PGI), which catalyses interconversions between fructose 6‐phosphate (F6P) and G6P. Under optimal growth conditions, the PGI reaction is strongly removed from equilibrium on the side of F6P resulting in low stromal G6P concentrations (Dietz, 1985; Gerhardt *et al*., 1987; Kruckeberg *et al*., 1989; Schleucher *et al*., 1999). Proposedly, low G6P concentrations restrict the anaplerotic flux (Sharkey & Weise, 2016). However, as the PGI reaction shifts towards equilibrium and G6P synthesis, anaplerotic flux into the CBC is believed to increase (Sharkey & Weise, 2016). This involves stromal glucose‐6‐phosphate dehydrogenase (G6PD), the first enzyme of the pentose phosphate pathway. In the light, G6PD is downregulated via redox regulation by thioredoxin (Née *et al*., 2009). However, downregulation can be allosterically reversed by increasing G6P concentrations (Cossar *et al*., 1984; Preiser *et al*., 2019).

*Phaseolus vulgaris* and *Spinacia oleracea* grown under optimal conditions exhibit pronounced D depletions at glucose H^2^ of leaf starch relative to sucrose (as mentioned in the previous section) (Schleucher *et al*., 1999). These depletions were attributed to the stromal PGI reaction being more strongly removed from equilibrium ([G6P]/[F6P] = 3.31) than the cytosolic PGI reaction (Dyson & Noltmann, 1968; Schleucher *et al*., 1999). Thus, as the stromal PGI reaction shifts towards equilibrium, H^2^ of stromal G6P and its derivatives including leaf starch and tree‐ring glucose may become less D‐depleted. Furthermore, G6PD catalyses the irreversible conversion of G6P to 6‐phosphogluconolactone and was shown to have a strong D isotope effect *in vitro* (*α* = 2.97) (Hermes *et al*., 1982). Thus, upregulation of the anaplerotic flux may cause D enrichments at H^1^ of stromal G6P and its derivatives.

As indicated by modelling, glucose H^1^ and H^2^ become D‐enriched as drought increases and *C*_a_ decreases beyond a change point (Tables 1, 2, 3; Fig. 3a–c). Increasing drought and decreasing *C*_a_ may cause decreasing *C*_i_ (as mentioned in a previous section), which may result in decreasing carbon assimilation. As carbon assimilation decreases below a change point, the stromal PGI reaction shifts towards equilibrium and G6P concentrations increase (Dietz, 1985). This may cause increasing G6PD activity and anaplerotic flux. Thus, expanding theory by Sharkey & Weise (2016), we propose anaplerotic flux into the CBC is upregulated in response to decreasing carbon assimilation, *C*_i_ and *C*_a_, and increasing drought beyond a change point.

Analysis of intramolecular ^13^C/^12^C ratios of the samples studied here has already provided evidence consistent with anaplerotic flux into the CBC at high VPD (Wieloch *et al*., 2018). These authors explained a strong common ^13^C signal at tree‐ring glucose C‐1 and C‐2 by anaplerotic flux changes. For shifts of the PGI reaction towards equilibrium, they predicted ^13^C/^12^C increases at C‐1 and C‐2 at a ratio of 2.25 and found a ratio of 2.74 (+1.35SE, −0.60SE) confirming their prediction. However, changes in sucrose‐to‐starch carbon partitioning would have the same effect. Furthermore, two recent studies report isotope and gas exchange evidence consistent with anaplerotic flux in *Helianthus annuus* at low *C*_i_ (Wieloch *et al*., 2021a, 2022a). That said, other mechanisms than those discussed here may contribute to the signals at tree‐ring glucose H^1^ and H^2^. This includes carbon flux into the oxidative branch of the cytosolic pentose phosphate pathway, shifts of the cytosolic PGI reaction and secondary isotope effects both in leaf and in tree‐ring cells.

### Metabolic fractionation signals at H^1^ and H^2^: a general phenomenon in C_3_ plants?

Cormier *et al*. (2018) grew six phylogenetically diverse angiosperms under varying *C*_a_. For leaf α‐cellulose, these authors reported average whole‐molecule *ε*_met_ increases of *c.* 20‰ in response to *C*_a_ decreases from 280 to 150 ppm, yet no significant change above 280 ppm. Hence, their observations are qualitatively in line with ours (Tables 1, 2, 3; Fig. 3a–c). However, our data show an average *ε*_met_ increase at tree‐ring glucose H^1^ and H^2^ of up to 150‰ (Fig. 2b). Assuming *c.* 40% heterotrophic hydrogen exchanges as observed at these positions in *Picea abies* (Augusti *et al*., 2006), we calculate an undiluted leaf‐level increase of *c.* 250‰ (150‰/60 × 100), which corresponds to a whole‐molecule increase of *c.* 70‰ (2 × 250‰/7). Thus, *ε*_met_ increases in the gymnosperm *Pinus nigra* are considerably larger than the *c.* 20‰ angiosperm average effect reported by Cormier *et al*. (2018). Additionally, whole‐molecule data have a limited interpretability (cf. ‘Introduction’) and effects reported by Cormier *et al*. (2018) may be located at either of the seven H‐C positions within α‐cellulose glucose. Thus, it remains uncertain whether the fractionation signals reported here occur generally in C_3_ plants.

### Conclusion

Stable isotope analysis on plant archives such as tree rings may convey information (*inter alia*) about plant acclimation and adaptation (e.g. to CO_2_ fertilisation), biosphere–atmosphere CO_2_ exchange and palaeoclimate trends. This long‐term perspective is inaccessible to manipulation and monitoring experiments yet crucial for understanding plant and Earth system functioning. The better we understand plant isotope fractionation, the more information can be retrieved from archives (at higher quality). The present paper and recent studies on metabolic fractionation (Wieloch et al., 2018, 2021a,d,c; Ladd *et al*., 2021) show there is much room for improvement. Thus, we recommend pushing the development of intramolecular isotope methodology.

## Author contributions

TW and JS conceived the study. TW led the research. TW, MG, HS, JS and IE collected and prepared the samples and acquired data. TW and JY analysed the data. TW developed theories about the origin of reported isotope signals. TW wrote the paper with input from AA, HS, JS and JY.

## Supporting information


**Notes S1** Statistical analyses.
**Notes S2** Grouping of annual *δ*D*_i_* patterns of tree‐ring glucose by HCA.
**Notes S3** Histograms of *ε*_met_.
**Notes S4** Bivariate relationships of *ε*_met_ with precipitation and atmospheric CO_2_ concentration.
**Notes S5** Contributions of *δ*D*_i_* to the variance in *δ*D_g_ after excluding data not affected by the fractionating metabolic processes.
**Notes S6** Contributions of *δ*D*_i_* to the variance in *δ*D_g_ after excluding data affected by the fractionating metabolic processes.
**Notes S7** Metabolic fractionation at the whole‐molecule level.
Please note: Wiley‐Blackwell are not responsible for the content or functionality of any Supporting Information supplied by the authors. Any queries (other than missing material) should be directed to the *New Phytologist* Central Office.

## Data Availability

The data that support the findings of this study are available from the corresponding author upon reasonable request.

## References

[nph18014-bib-0001] Abtew W , Melesse AM . 2013. Chapter 5 – vapor pressure calculation methods. Evaporation and evapotranspiration. Dordrecht, the Netherlands: Springer, 53–62.

[nph18014-bib-0002] Augusti A , Betson TR , Schleucher J . 2006. Hydrogen exchange during cellulose synthesis distinguishes climatic and biochemical isotope fractionations in tree rings. New Phytologist 172: 490–499. 17083679 10.1111/j.1469-8137.2006.01843.x

[nph18014-bib-0003] Betson TR , Augusti A , Schleucher J . 2006. Quantification of deuterium isotopomers of tree‐ring cellulose using Nuclear Magnetic Resonance. Analytical Chemistry 78: 8406–8411. 17165833 10.1021/ac061050a

[nph18014-bib-0004] Cernusak LA , Barbour MM , Arndt SK , Cheesman AW , English NB , Feild TS , Helliker BR , Holloway‐Phillips MM , Holtum JAM , Kahmen A *et al*. 2016. Stable isotopes in leaf water of terrestrial plants. Plant, Cell & Environment 39: 1087–1102. 10.1111/pce.12703 26715126

[nph18014-bib-0005] Chen Y , Helliker BR , Tang X , Li F , Zhou Y , Song X . 2020. Stem water cryogenic extraction biases estimation in deuterium isotope composition of plant source water. Proceedings of the National Academy of Sciences, USA 117: 33345–33350. 10.1073/pnas.2014422117 PMC7776815 33318208

[nph18014-bib-0006] Cormier M‐A , Werner RA , Sauer PE , Gröcke DR , Leuenberger MC , Wieloch T , Schleucher J , Kahmen A . 2018. ^2^H‐fractionations during the biosynthesis of carbohydrates and lipids imprint a metabolic signal on the *δ* ^2^H values of plant organic compounds. New Phytologist 218: 479–491. 29460486 10.1111/nph.15016

[nph18014-bib-0007] Cossar JD , Rowell P , Stewart WDP . 1984. Thioredoxin as a modulator of glucose‐6‐phosphate dehydrogenase in a N_2_‐fixing cyanobacterium. Microbiology 130: 991–998.

[nph18014-bib-0008] Craig H , Gordon LI . 1965. Deuterium and oxygen 18 variations in the ocean and the marine atmosphere. In: Tongiorgi E , ed. Stable isotopes in oceanographic studies and paleotemperatures. Pisa, Italy: Consiglio nazionale delle ricerche, Laboratorio di geologia nucleare, 9–130.

[nph18014-bib-0009] Dietz K‐J . 1985. A possible rate‐limiting function of chloroplast hexosemonophosphate isomerase in starch synthesis of leaves. Biochimica et Biophysica Acta 839: 240–248.

[nph18014-bib-0010] Dyson JE , Noltmann EA . 1968. The effect of pH and temperature on the kinetic parameters of phosphoglucose isomerase. Participation of histidine and lysine in a proposed dual function mechanism. Journal of Biological Chemistry 243: 1401–1414. 5647261

[nph18014-bib-0011] Ehlers I , Augusti A , Betson TR , Nilsson MB , Marshall JD , Schleucher J . 2015. Detecting long‐term metabolic shifts using isotopomers: CO_2_‐driven suppression of photorespiration in C_3_ plants over the 20^th^ century. Proceedings of the National Academy of Sciences, USA 112: 15585–15590. 10.1073/pnas.1504493112 PMC4697390 26644588

[nph18014-bib-0012] Evans JR , Farquhar GD , Sharkey TD , Berry JA . 1986. Carbon isotope discrimination measured concurrently with gas exchange to investigate CO_2_ diffusion in leaves of higher plants. Australian Journal of Plant Physiology 13: 281–292.

[nph18014-bib-0013] Farquhar GD , Cernusak LA , Barnes B . 2007. Heavy water fractionation during transpiration. Plant Physiology 143: 11–18. 17210909 10.1104/pp.106.093278 PMC1761995

[nph18014-bib-0014] Farquhar GD , O’Leary MH , Berry JA . 1982. On the relationship between carbon isotope discrimination and the intercellular carbon dioxide concentration in leaves. Australian Journal of Plant Physiology 9: 121–137.

[nph18014-bib-0015] Flanagan LB , Comstock JP , Ehleringer JR . 1991. Comparison of modeled and observed environmental influences on the stable oxygen and hydrogen isotope composition of leaf water in *Phaseolus vulgaris* L. Plant Physiology 96: 588–596. 16668226 10.1104/pp.96.2.588 PMC1080811

[nph18014-bib-0016] Fong Y , Huang Y , Gilbert PB , Permar SR . 2017. chngpt: threshold regression model estimation and inference. BMC Bioinformatics 18: 454. 29037149 10.1186/s12859-017-1863-x PMC5644082

[nph18014-bib-0017] Frank DC , Poulter B , Saurer M , Esper J , Huntingford C , Helle G , Treydte K , Zimmermann NE , Schleser G h , Ahlström A *et al*. 2015. Water‐use efficiency and transpiration across European forests during the Anthropocene. Nature Climate Change 5: 579–583.

[nph18014-bib-0018] Gerhardt R , Stitt M , Heldt HW . 1987. Subcellular metabolite levels in spinach leaves: regulation of sucrose synthesis during diurnal alterations in photosynthetic partitioning. Plant Physiology 83: 399–407. 16665257 10.1104/pp.83.2.399 PMC1056369

[nph18014-bib-0019] Gray J , Song SJ . 1984. Climatic implications of the natural variations of D/H ratios in tree ring cellulose. Earth and Planetary Science Letters 70: 129–138.

[nph18014-bib-0020] Hermes JD , Roeske CA , O’Leary MH , Cleland WW . 1982. Use of multiple isotope effects to determine enzyme mechanisms and intrinsic isotope effects. Malic enzyme and glucose 6‐phosphate dehydrogenase. Biochemistry 21: 5106–5114. 7138850 10.1021/bi00263a040

[nph18014-bib-0021] Kahmen A , Hoffmann B , Schefuß E , Arndt SK , Cernusak LA , West JB , Sachse D . 2013. Leaf water deuterium enrichment shapes leaf wax n‐alkane *δ*D values of angiosperm plants II: observational evidence and global implications. Geochimica et Cosmochimica Acta 111: 50–63.

[nph18014-bib-0022] Köhler IH , Macdonald AJ , Schnyder H . 2016. Last‐century increases in intrinsic water‐use efficiency of grassland communities have occurred over a wide range of vegetation composition, nutrient inputs, and soil pH. Plant Physiology 170: 881–890. 26620525 10.1104/pp.15.01472 PMC4734565

[nph18014-bib-0023] Kruckeberg AL , Neuhaus HE , Feil R , Gottlieb LD , Stitt M . 1989. Decreased‐activity mutants of phosphoglucose isomerase in the cytosol and chloroplast of *Clarkia xantiana* . Biochemical Journal 261: 457–467. 2775228 10.1042/bj2610457 PMC1138848

[nph18014-bib-0024] Ladd SN , Nelson DB , Bamberger I , Daber LE , Kreuzwieser J , Kahmen A , Werner C . 2021. Metabolic exchange between pathways for isoprenoid synthesis and implications for biosynthetic hydrogen isotope fractionation. New Phytologist 231: 1708–1719. 34028817 10.1111/nph.17510

[nph18014-bib-0025] Leal S , Eamus D , Grabner M , Wimmer R , Cherubini P . 2008. Tree rings of *Pinus nigra* from the Vienna basin region (Austria) show evidence of change in climatic sensitivity in the late 20^th^ century. Canadian Journal of Forest Research 38: 744–759.

[nph18014-bib-0026] Loader NJ , Walsh RP , Robertson I , Bidin K , Ong RC , Reynolds G , McCarroll D , Gagen M , Young GH . 2011. Recent trends in the intrinsic water‐use efficiency of ringless rainforest trees in Borneo. Philosophical Transactions of the Royal Society of London 366: 3330–3339. 22006972 10.1098/rstb.2011.0037 PMC3179630

[nph18014-bib-0027] Martin GJ , Zhang BL , Naulet N , Martin ML . 1986. Deuterium transfer in the bioconversion of glucose to ethanol studied by specific labeling at the natural abundance level. Journal of the American Chemical Society 108: 5116–5122.

[nph18014-bib-0028] McKee TB , Doesken NJ , Kleist J . 1993. The relationship of drought frequency and duration to time scales. In: Proceedings of the 8^th^ conference on applied climatology. Anaheim, CA, USA: American Meteorological Society, 179–184.

[nph18014-bib-0029] Melander LCS , Saunders WH . 1980. Reaction rates of isotopic molecules. New York, NY, USA: John Wiley & Sons.

[nph18014-bib-0030] Née G , Zaffagnini M , Trost P , Issakidis‐Bourguet E . 2009. Redox regulation of chloroplastic glucose‐6‐phosphate dehydrogenase: a new role for f‐type thioredoxin. FEBS Letters 583: 2827–2832. 19631646 10.1016/j.febslet.2009.07.035

[nph18014-bib-0031] Peñuelas J , Canadell JG , Ogaya R . 2011. Increased water‐use efficiency during the 20^th^ century did not translate into enhanced tree growth. Global Ecology and Biogeography 20: 597–608.

[nph18014-bib-0032] Peñuelas J , Filella I . 2003. Deuterium labelling of roots provides evidence of deep water access and hydraulic lift by *Pinus nigra* in a Mediterranean forest of NE Spain. Environmental and Experimental Botany 49: 201–208.

[nph18014-bib-0033] Preiser AL , Fisher N , Banerjee A , Sharkey TD . 2019. Plastidic glucose‐6‐phosphate dehydrogenases are regulated to maintain activity in the light. Biochemical Journal 476: 1539–1551. 31092702 10.1042/BCJ20190234 PMC6626494

[nph18014-bib-0034] Quick P , Siegl G , Neuhaus E , Feil R , Stitt M . 1989. Short‐term water stress leads to a stimulation of sucrose synthesis by activating sucrose‐phosphate synthase. Planta 177: 535–546. 24212496 10.1007/BF00392622

[nph18014-bib-0035] Quick WP , Chaves MM , Wendler R , David M , Rodrigues ML , Passaharinho JA , Pereira JS , Adcock MD , Leegood RC , Stitt M . 1992. The effect of water stress on photosynthetic carbon metabolism in four species grown under field conditions. Plant, Cell & Environment 15: 25–35.

[nph18014-bib-0036] Roden JS , Ehleringer JR . 1999a. Observations of hydrogen and oxygen isotopes in leaf water confirm the Craig‐Gordon model under wide‐ranging environmental conditions. Plant Physiology 120: 1165–1174. 10444100 10.1104/pp.120.4.1165 PMC59350

[nph18014-bib-0037] Roden JS , Ehleringer JR . 1999b. Hydrogen and oxygen isotope ratios of tree‐ring cellulose for riparian trees grown long‐term under hydroponically controlled environments. Oecologia 121: 467–477. 28308356 10.1007/s004420050953

[nph18014-bib-0038] Roden JS , Ehleringer JR . 2000. Hydrogen and oxygen isotope ratios of tree ring cellulose for field‐grown riparian trees. Oecologia 123: 481–489. 28308756 10.1007/s004420000349

[nph18014-bib-0039] Roden JS , Lin G , Ehleringer JR . 2000. A mechanistic model for interpretation of hydrogen and oxygen isotope ratios in tree‐ring cellulose. Geochimica et Cosmochimica Acta 64: 21–35.

[nph18014-bib-0040] Rose IA , O’Connell EL . 1961. Intramolecular hydrogen transfer in the phosphoglucose isomerase reaction. Journal of Biological Chemistry 236: 3086–3092. 14493830

[nph18014-bib-0041] Ross GJ . 2015. Parametric and nonparametric sequential change detection in R: the cpm package. Journal of Statistical Software 66: 1–19.

[nph18014-bib-0042] Sade N , Gebremedhin A , Moshelion M . 2012. Risk‐taking plants: anisohydric behavior as a stress‐resistance trait. Plant Signaling & Behavior 7: 767–770. 22751307 10.4161/psb.20505 PMC3583960

[nph18014-bib-0043] Saurer M , Spahni R , Frank DC , Joos F , Leuenberger M , Loader NJ , McCarroll D , Gagen M , Poulter B , Siegwolf RTW *et al*. 2014. Spatial variability and temporal trends in water‐use efficiency of European forests. Global Change Biology 20: 3700–3712. 25156251 10.1111/gcb.12717

[nph18014-bib-0044] Schleucher J . 1998. Intramolecular deuterium distributions and plant growth conditions. In: Griffiths H , ed. Stable isotopes – integration of biological, ecological and geochemical processes. Oxford, UK: Bios Scientific Publishers, 63–73.

[nph18014-bib-0045] Schleucher J , Vanderveer P , Markley JL , Sharkey TD . 1999. Intramolecular deuterium distributions reveal disequilibrium of chloroplast phosphoglucose isomerase. Plant, Cell & Environment 22: 525–533.

[nph18014-bib-0046] Schmidt HL , Robins RJ , Werner RA . 2015. Multi‐factorial *in vivo* stable isotope fractionation: causes, correlations, consequences and applications. Isotopes in Environmental and Health Studies 51: 155–199. 25894429 10.1080/10256016.2015.1014355

[nph18014-bib-0047] Schmidt H‐L , Werner RA , Eisenreich W . 2003. Systematics of ^2^H patterns in natural compounds and its importance for the elucidation of biosynthetic pathways. Phytochemistry Reviews 2: 61–85.

[nph18014-bib-0048] Sharkey TD , Berry JA , Raschke K . 1985. Starch and sucrose synthesis in *Phaseolus vulgaris* as affected by light, CO_2_, and abscisic acid. Plant Physiology 77: 617–620. 16664108 10.1104/pp.77.3.617 PMC1064574

[nph18014-bib-0049] Sharkey TD , Weise SE . 2016. The glucose 6‐phosphate shunt around the Calvin‐Benson cycle. Journal of Experimental Botany 67: 4067–4077. 26585224 10.1093/jxb/erv484

[nph18014-bib-0050] Speer JH . 2010. Fundamentals of tree‐ring research. Tucson, AZ, USA: The University of Arizona Press.

[nph18014-bib-0051] Stokes A , Fourcaud T , Hruska J , Cermák J , Nadyezdhina N , Nadyezhdin V , Praus L . 2002. An evaluation of different methods to investigate root system architecture of urban trees *in situ*: I. ground‐penetrating radar. Journal of Arboriculture 28: 2–10.

[nph18014-bib-0052] Vassey TL , Sharkey TD . 1989. Mild water stress of *Phaseolus vulgaris* plants leads to reduced starch synthesis and extractable sucrose phosphate synthase activity. Plant Physiology 89: 1066–1070. 16666665 10.1104/pp.89.4.1066 PMC1055976

[nph18014-bib-0053] Vicente‐Serrano SM , Beguería S , López‐Moreno JI . 2010. A multiscalar drought index sensitive to global warming: the standardized precipitation evapotranspiration index. Journal of Climate 23: 1696–1718.

[nph18014-bib-0054] Waterhouse JS , Switsur VR , Barker AC , Carter AHC , Robertson I . 2002. Oxygen and hydrogen isotope ratios in tree rings: how well do models predict observed values? Earth and Planetary Science Letters 201: 421–430.

[nph18014-bib-0055] Wells N , Goddard S , Hayes MJ . 2004. A self‐calibrating Palmer drought severity index. Journal of Climate 17: 2335–2351.

[nph18014-bib-0057] Wieloch T , Augusti A , Schleucher J . 2021a. Anaplerotic flux into the Calvin‐Benson cycle. Integration in carbon and energy metabolism of *Helianthus annuus* . bioRxiv 2021.07.30.454461.

[nph18014-bib-0056] Wieloch T , Augusti A , Schleucher J . 2022a. Anaplerotic flux into the Calvin‐Benson cycle. Hydrogen isotope evidence for *in vivo* occurrence in C_3_ metabolism. New Phytologist. 10.1111/nph.17957. PMC9305100 35020197

[nph18014-bib-0058] Wieloch T , Ehlers I , Yu J , Frank D , Grabner M , Gessler A , Schleucher J . 2018. Intramolecular ^13^C analysis of tree rings provides multiple plant ecophysiology signals covering decades. Scientific Reports 8: 5048. 29567963 10.1038/s41598-018-23422-2 PMC5864875

[nph18014-bib-0059] Wieloch T , Sharkey TD , Werner RA , Schleucher J . 2022b. Intramolecular carbon isotope signals reflect metabolite allocation in plants. Journal of Experimental Botany. 10.1093/jxb/erac028. PMC9015809 35084456

[nph18014-bib-0060] Wieloch T , Werner RA , Schleucher J . 2021b. Carbon flux around leaf‐cytosolic glyceraldehyde‐3‐phosphate dehydrogenase introduces a ^13^C signal in plant glucose. Journal of Experimental Botany 72: 7136–7144. 34223885 10.1093/jxb/erab316 PMC8547152

[nph18014-bib-0061] Yakir D , DeNiro MJ . 1990. Oxygen and hydrogen isotope fractionation during cellulose metabolism in *Lemna gibba* L. Plant Physiology 93: 325–332. 16667454 10.1104/pp.93.1.325 PMC1062506

