# Supplementary material for: Metabolism is a major driver of hydrogen isotope fractionation recorded in tree‐ring glucose of Pinus nigra
Source: New Phytol. 2022 Feb 26;234(2):449–61. doi: 10.1111/nph.18014 (PMC9306475; doi:10.1111/nph.18014)
Supplement: Supplementary file 1 — Notes S1 Statistical analyses. Notes S2 Grouping of annual δD i patterns of tree‐ring glucose by HCA. Notes S3 Histograms of ε met. Notes S4 Bivariate relationships of ε met with precipitation and atmospheric CO2 concentration. Notes S5 Contributions of δD i to the variance in δDg after excluding data not affected by the fractionating metabolic processes. Notes S6 Contributions of δD i to the variance in δDg after excluding data affected by the fractionating metabolic processes. Notes S7 Metabolic fractionation at the whole‐molecule level. Please note: Wiley‐Blackwell are not responsible for the content or functionality of any Supporting Information supplied by the authors. Any queries (other than missing material) should be directed to the New Phytologist Central Office. [file NPH-234-449-s001.pdf]

Supporting information - Metabolism is a major driver of hydrogen isotope fractionation recorded in tree-ring glucose of *Pinus nigra*

Thomas Wieloch, Michael Grabner, Angela Augusti, Henrik Serk, Ina Ehlers, Jun Yu, Jürgen Schleucher (Accepted: 24 January 2022)

Notes S1. Statistical analyses

Hierarchical cluster analysis was performed using the R function hclust() of the STATS package on z-scores of δD_i using Euclidean distances and Ward's fusion criterion for cluster formation (n=7*31). Mood's median tests were performed using the R function median_test() of the COIN package. Batch change point analysis based on the non-parametric Mann-Whitney-Wilcoxon test was performed using the R function detectChangePointBatch() of the CPM package (Ross, 2015). Multiple linear regression modelling was performed using the R function lm() of the STATS package. Change point regression modelling (step type) was performed using the R function chngptm() of the CHNGPT package (Fong *et al.*, 2017). Statistical significances of the change point and the change point model were calculated using the R functions chngpt.test() of the CHNGPT package and lrtest() of the LMTEST package, respectively.

Notes S2. Grouping of annual δD_i patterns of tree-ring glucose by HCA

Metabolic fractionations affect specific intramolecular H-C positions and thus introduce intramolecular δD_i patterns (Figs. 1, 2b, and S2b). To identify annual patterns that were similarly/differently modified by metabolic fractionation, we performed a Hierarchical Cluster Analysis (HCA) on annual δD_i patterns (Fig. S2a). We found two groups of δD_i patterns: a high-value and a low-value group according to δD_1 and δD_2 values (Figs. 2b, and S2b). Please note that the population to which our data belong must be continuously distributed despite the apparent grouping and the gap in δD_2 in the ≈200 to 250‰ range (Figs. S2a, and S2b). This is because tree rings record ecophysiological information continuously over the course of growing seasons, i.e., the temporal impact of metabolic fractionations at H^1 and H^2 principally varies in a continuous way. Nevertheless, the apparent grouping in our data is convenient to investigate causes of high and low δD_1 and δD_2 values. Please note that if our dataset had been continuously distributed, we could still have arbitrarily separated it into low- and high-value groups and investigated underlying causes. However, in the present case, HCA separates the data for us.

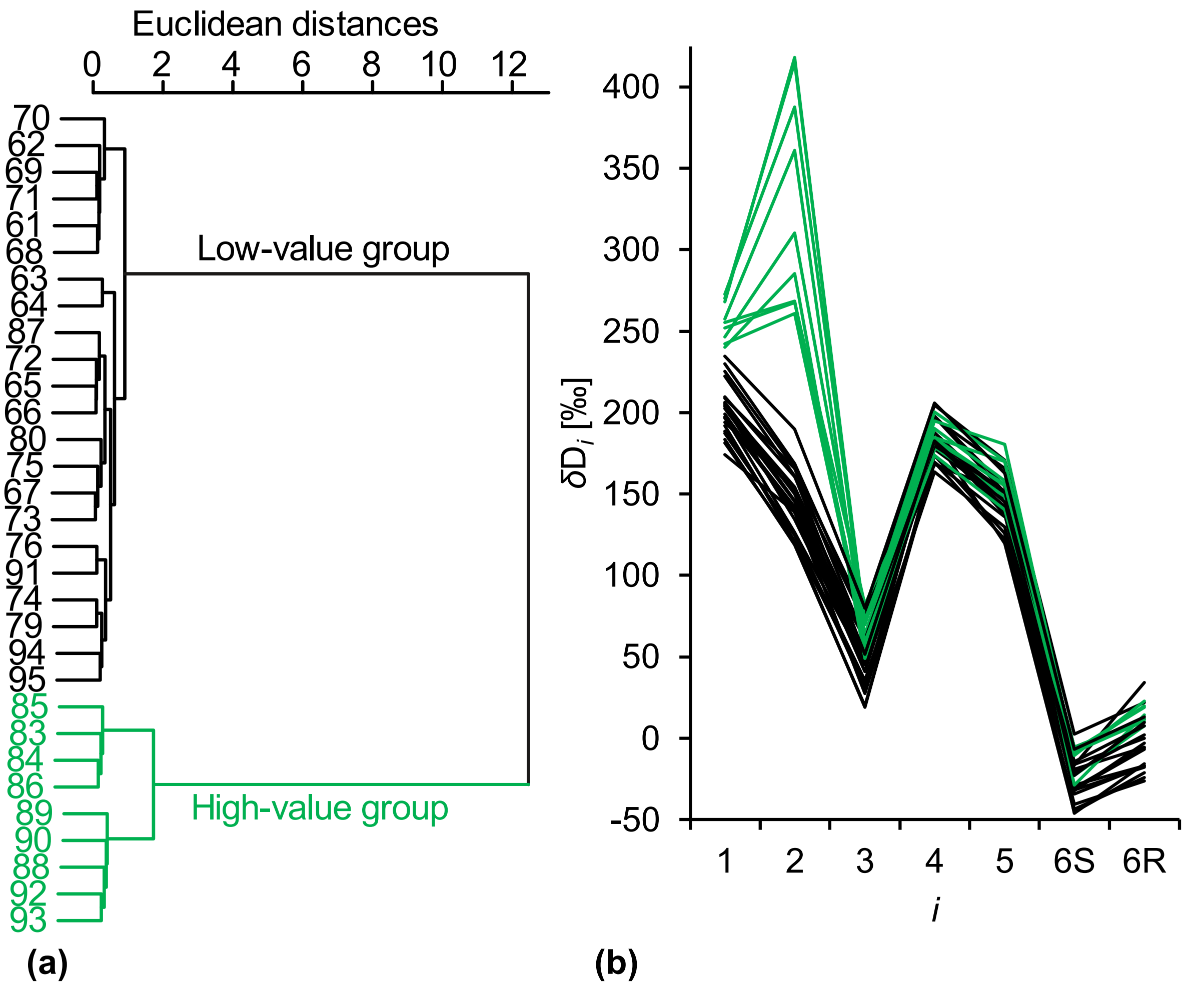

Figure S2 (a) Grouping of annual δD_i patterns by Hierarchical Cluster Analysis (HCA). **(b)** Annual δD_i patterns. δD_i denotes D abundances at intramolecular H-C positions in tree-ring glucose. Data were acquired for tree-ring glucose of *Pinus nigra* laid down from 1961 to 1995 at a site in the Vienna basin (±*SE*=5.4‰, $n \geq 3$). Prior to HCA, outliers were replaced by timeseries averages. Data reference: Average D abundance of the methyl-group hydrogens of the glucose derivative used for NMR measurements. Figure S2b shows discrete data. Lines used to guide the eye.

Notes S3. Histograms of ε_{met}

Figures S3a, and b show histograms of ε_{met} including all data, 1961 to 1995, and data of 1983 to 1995, respectively. The dataset including all data clearly does not follow a normal distribution. By contrast, there are no indications that the dataset including only years in which groundwater levels were low deviates from a normal distribution (1983 to 1995, Fig. 3a).

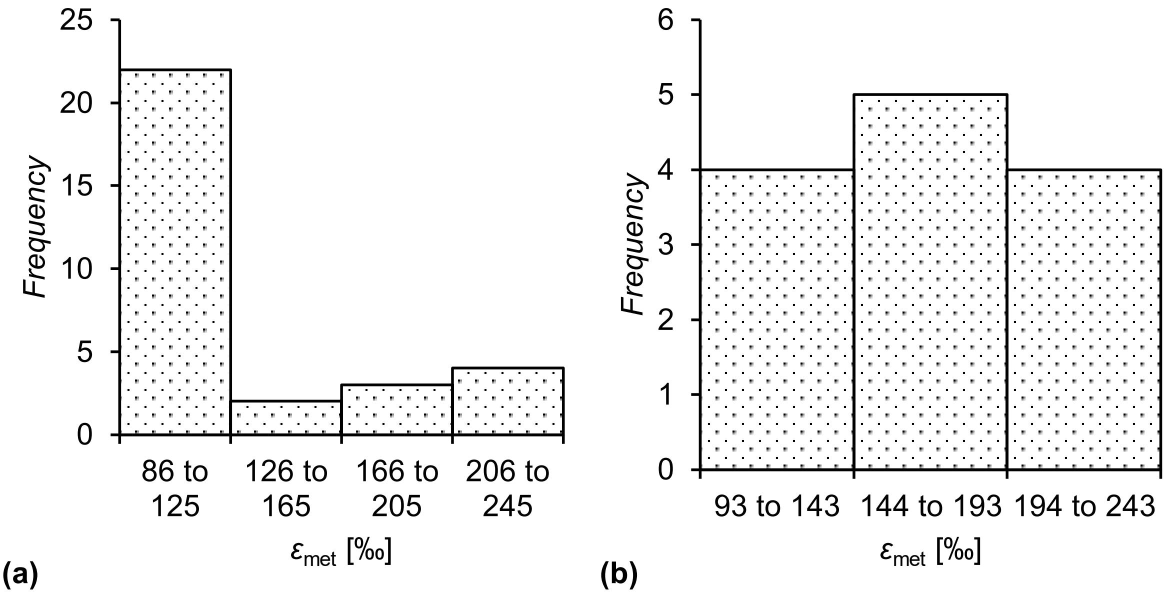

Figure S3 (a) Histogram of average metabolic fractionation at glucose H^1 and H^2, ε_{met}, including all data, 1961 to 1995 (*n*=31). **(b)** Histogram of ε_{met} including data of 1983 to 1995 corresponding to years with low groundwater level (*n*=13, Fig. 3a). Data for the calculation of ε_{met} were acquired for tree-ring glucose of *Pinus nigra* laid down from 1961 to 1995 at a site in the Vienna basin (±*SE*=3.5‰, *n*≥3).

Notes S4. Bivariate relationships of ε_{met} with precipitation and atmospheric CO_2 concentration

Figures S4a, and b show bivariate relationships of ε_{met} with March to July precipitation, *PRE*, and annual atmospheric CO_2 concentrations, C_a, respectively, for two groups of data. While the first group includes data of 1961 to 1982 corresponding to high groundwater storage (black), the second group includes data of 1983 to 1995 corresponding to low groundwater storage (blue, Fig. 3a). Linear modelling shows that relationships of ε_{met} with both environmental parameters are group specific. While the first group shows no significant relationships (ε_{met} ~ *PRE*: R^2=0.21, p>.05, n=18; ε_{met} ~ C_a: R^2=0.01, p>.7, n=18), the second group shows significant negative relationships (ε_{met} ~ *PRE*: R^2=0.71, p<0.001, n=13; ε_{met} ~ C_a: R^2=0.54, p<.01, n=13). Note that low ε_{met} values can occur under low groundwater storage (Fig. 3b, 1987, 1991, 1994, and 1995). This is explained by high precipitation during spring and summer and high atmospheric CO_2 concentrations (Figs. S4a, and b, filled circles).

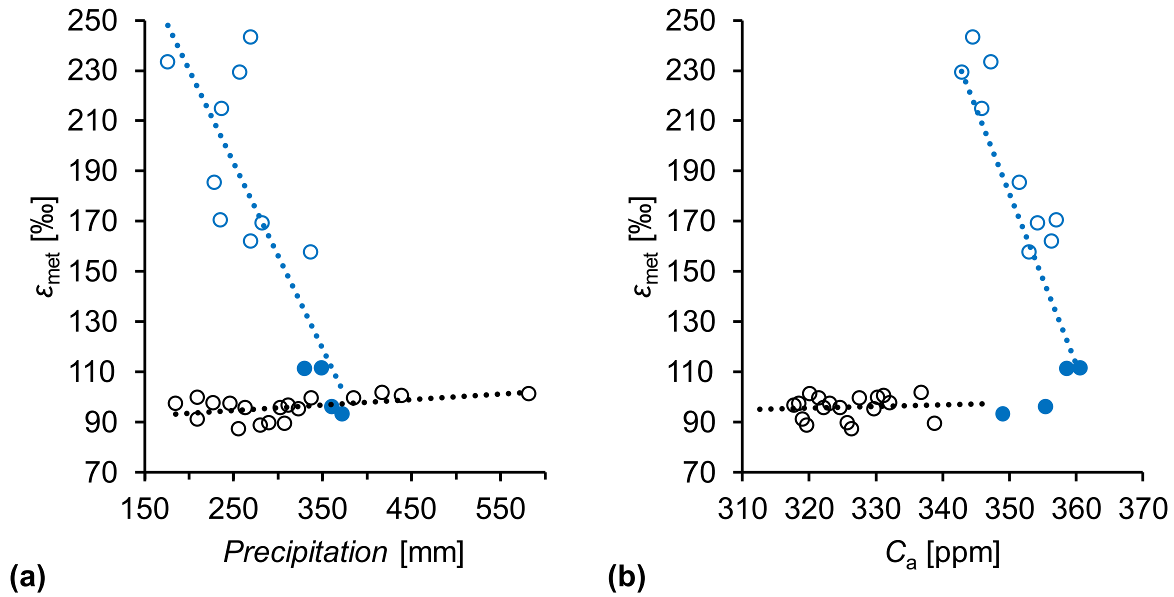

Figure S4 (a) Relationship between average metabolic fractionation at glucose H[1] and H[2], ε_{met}, and March to July precipitation. **(b)** Relationship between ε_{met} and annual atmospheric CO_2 concentration, C_a. Black and blue circles: Data of years with high groundwater storage, 1961 to 1982, and low groundwater storage, 1983 to 1995, respectively (Fig. 3a). Filled circles: Data of 1987, 1991, 1994, and 1995. Dotted black and blue lines: Trendlines pertaining to datasets of the same colour. Data for the calculation of ε_{met} were acquired for tree-ring glucose of *Pinus nigra* laid down from 1961 to 1995 at a site in the Vienna basin (±*SE*=3.5‰, *n*≥3).

Notes S5. Contributions of δD_i to the variance in δD_g after excluding data not affected by the fractionating metabolic processes

Metabolic fractionations in δD_1 and δD_2 have a strong weight on whole-molecule D variability, δD_g. To assess this weight exclusively for years with upregulated fractionating metabolic processes, we repeated the variance partitioning on corresponding data but exclude 1988 and 1990 because of data gaps as result of the outlier analysis (1983-1986, 1989, 1992, and 1993; Fig. 2c). We found that δD_1 and δD_2 together account for 86.8% of the variance in δD_g. By contrast, δD_3 to δD_5 each account for 6.3% on average. Interestingly, δD_{6S} and δD_{6R} reduce the variability of δD_g by -2.8% on average. Assuming the variability in δD_3 to δD_5 reflects the combined influence of known fractionation processes affecting all δD_i, such as leaf water D enrichment, metabolic fractionations in δD_1 and δD_2 together account for 74.2% of the variance in δD_g (86.8%-2*6.3%).

Notes S6. Contributions of δD_i to the variance in δD_g after excluding data affected by the fractionating metabolic processes

Metabolic processes have strong effects on δD_1, δD_2, and δD_g (Figs. 1, and 2). After excluding years affected by these processes from the variance partitioning analysis (1983 to 1995), all δD_i exhibit similar degrees of variance and contribute similarly to δD_g (Fig. S6).

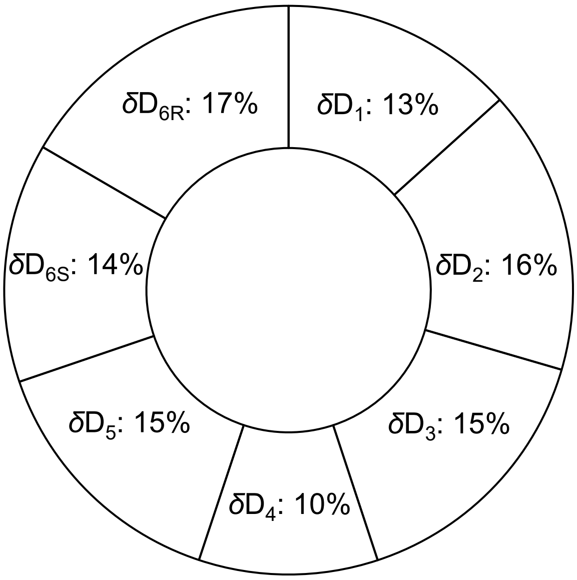

Figure S6 Percentage contributions of δD_i to the variance of δD_g. δD_i and δD_g denote timeseries of D abundances at intramolecular H-C positions in glucose and of the whole molecule, respectively. Data were acquired for tree-ring glucose of *Pinus nigra* laid down from 1961 to 1982 at a site in the Vienna basin (δD_i: ±SE=5.5‰, n≥3; δD_g: ±SE=3.7‰, n≥3). Outliers were removed prior to analysis. The analysis is based on years without missing data (n=8*14). Data reference: Average D abundance of the methyl-group hydrogens of the glucose derivative used for NMR measurements.

Notes S7. Metabolic fractionation at the whole-molecule level

Within this paragraph, the term ‘metabolic fractionation’ refers to metabolic fractionation at glucose H^1 and H^2.

Variability in δD_g is predominantly controlled by metabolic fractionation (Fig. 2d). Since δD_g can be measured by high-throughput isotope ratio mass spectrometry (a technique accessible to numerous laboratories), we will now investigate possibilities to (i) identify δD_g datasets affected

by metabolic fractionation, (ii) separate δD_g datapoints affected by metabolic fractionation from other datapoints, and (iii) retrieve information from δD_g about metabolic fractionation.

(i) Metabolic fractionation caused occasional δD_g increases above normal δD_g values but never δD_g decreases (green dots in Fig. S7a). Consequently, the δD_g distribution is asymmetrical with a moderate positive skew, has increased variability, and is nearly significantly different from normality (Fig. S7c; *skewness*=0.55, *range*=83.2‰, *SD*=23.8‰; Shapiro-Wilk normality test: W=0.93616, p=0.12). After excluding data affected by metabolic fractionation, the δD_g distribution is approximately symmetrical, has lower variability, and is not significantly different from normality (Fig. S7d; *skewness*=0.32, *range*=47.6‰, *SD*=13.5‰; Shapiro-Wilk normality test: W=0.95866, p=0.58). Furthermore, we found a change point in the complete δD_g timeseries (non-parametric Mann-Whitney-Wilcoxon test: p<.001, n=25) which corresponds to the change point in ε_{met} and marks the onset of a period with conditions favourable for upregulations of the fractionating metabolic processes (main text, '*Step 1*'). Thus, both visual inspection of the δD_g distribution and statistical tests indicate effects by metabolic fractionation.

Our δD_g dataset is relatively small (n=25) and, therefore, not an ideal approximation of the underlying probability distribution. Theoretically, we would expect a bimodal distribution. Data not affected by metabolic fractionation (black dots in Fig. S7a) would be represented by a low-value peak in the histogram. Data affected by metabolic fractionation (green dots in Fig. S7a) would be represented by a high-value peak adjacent to the low-value peak. The relative height of these peaks would depend on the relative frequency of long-term drought events (groundwater depletions below the critical level).

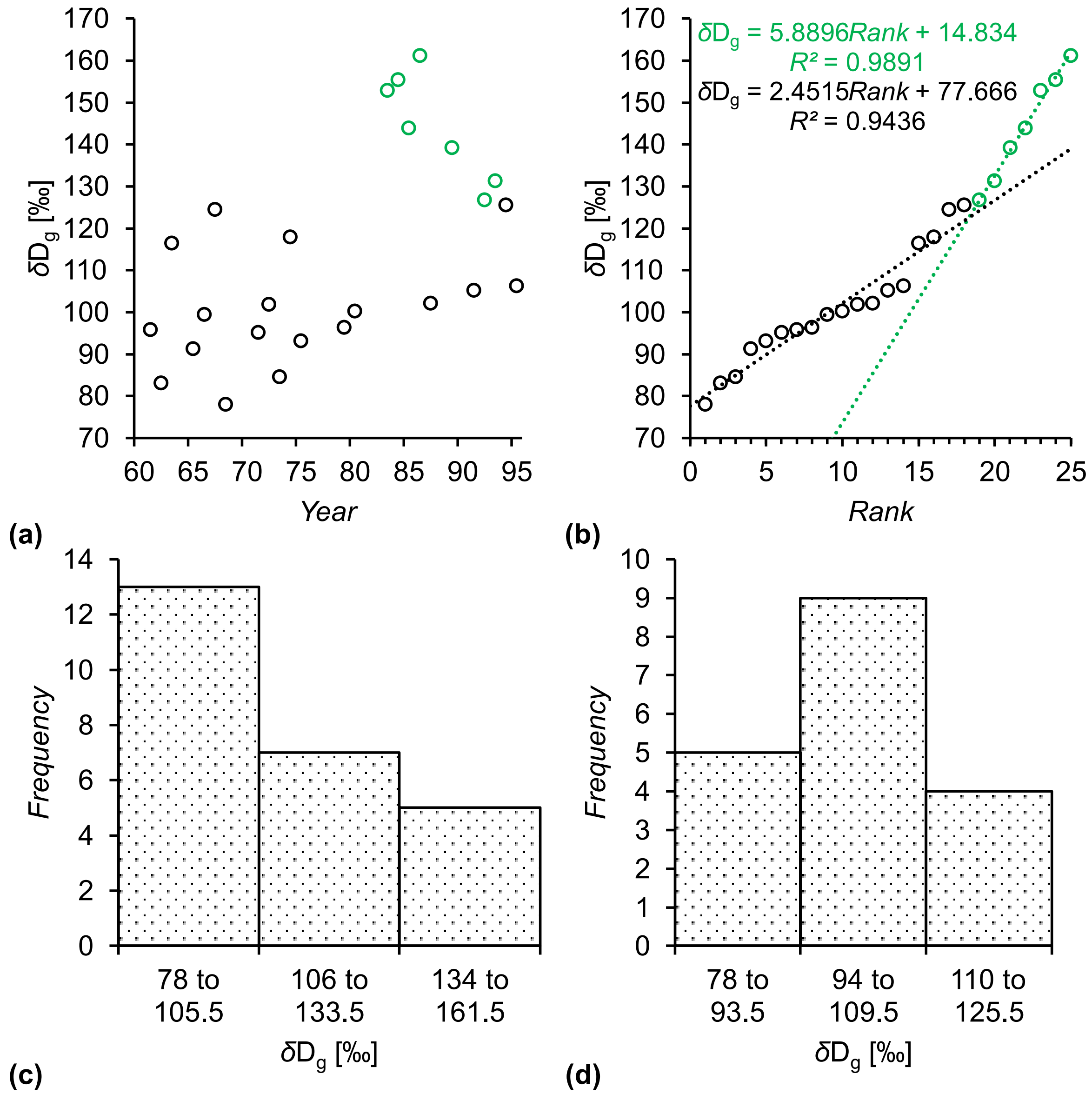

Figure S7 (a) Timeseries of whole-molecule deuterium abundance, δD_g. Green and black dots, data affected and not affected by metabolic fractionation at glucose H^1 and H^2, respectively (n=7, and n=18). **(b)** δD_g ranked according to value from low to high. Green and black lines, trendlines pertaining to data affected and not affected by metabolic fractionation at glucose H^1 and H^2, respectively. **(c)** Histogram of δD_g including all data (n=25, black and green dots in Fig. S7a). **(d)** Histogram of δD_g excluding data affected by metabolic fractionations at glucose H^1 and H^2 (n=18, black dots in Fig. S7a). Data were acquired for tree-ring glucose of *Pinus nigra* laid down from 1961 to 1995 at a site in the Vienna basin (±*SE*=3.4‰, $n \geq 3$). Outliers were removed prior to analysis. Data reference: Average D abundance of the methyl-group hydrogens of the glucose derivative used for NMR measurements.

(ii) Figure S7a shows δD_g as function of time with green dots representing data affected by metabolic fractionation (*cf.* Fig. 2c). Without colour coding, a clear separation between datapoints affected by metabolic fractionation and other datapoints is not feasible. Figure S7b shows the same data ranked by value from low to high. Data affected by metabolic fractionation have the highest ranks and are sitting neatly on a line (green line, R^2=0.99, n=7). The slope of this line is 2.4 times steeper than the slope of the line pertaining to data not affected by metabolic fractionation (black line, R^2=0.94, n=18). This may enable δD_g data separation yet not with high confidence. For instance, without colour coding, it is unclear whether the four datapoints before the green datapoints were also affected by metabolic fractionation. Furthermore, if the number of data affected by metabolic fractionation was 2.4 times higher (n≈17), both lines would have the same slope.

(iii) A change point model explains most of the variance in ε_{met} (main text, '*Model 2*', R^2=0.94, $p<10^{-15}$, n=31, Eq. 7), and all explanatory variables contribute significantly to this model (Table 3). While the same change point model explains a significant fraction of the variance in δD_g (R^2=0.76, $p<10^{-5}$, n=25, Eq. 7), most explanatory variables do not contribute significantly (Table S7). Thus, at the level of δD_g, the environmental dependences of the fractionating metabolic processes are insufficiently constraint for interpretation.

Table S7 Estimated coefficients of the δD_g change point model.

Coefficient	Estimate	SE*	Lower 95% CI	Upper 95% CI	p-value*
β_1	638.12	328.99	-246.67	1042.99	0.05
β_2	-0.21463	0.09973	-0.40477	-0.01382	<.05
β_3	-1.2620	0.9331	-2.4785	1.1794	0.2
β_4	-562.80	730.68	-2260.27	604.01	0.4
β_5	0.18721	0.13934	-0.07207	0.47415	0.2
β_6	1.3598	2.3345	-2.0441	7.1071	0.6
**e**	-0.70310	0.23832	-0.85880	0.07540	<.001

A change point model was fitted to measured whole-molecule deuterium abundances, δD_g (R^2=0.76, $p<10^{-5}$, n=25, Eq. 7) (Fong *et al.*, 2017). Data were acquired for tree-ring glucose of *Pinus nigra* laid down from 1961 to 1995 at a site in the Vienna basin (±*SE*=3.4‰, $n\geq3$). β_1 to β_6, and e denote model coefficients (Eq. 7). SE and CI denote the standard error and confidence interval, respectively. Asterisks mark estimations which assume that bootstrap sampling followed a normal distribution.

References

Fong Y, Huang Y, Gilbert PB, Permar SR. **2017**. chngpt: threshold regression model estimation and inference. *BMC Bioinformatics* **18**: 454.

Ross GJ. 2015. Parametric and nonparametric sequential change detection in R: The cpm package. *Journal of Statistical Software* **66**: 1–19.
